# Asymmetric Electrolytes Govern Tetrahydroxozincate Dynamics for Stable Alkaline Zinc Batteries

**DOI:** 10.1002/anie.202524438

**Published:** 2026-02-09

**Authors:** Xianhong Chen, Yang Wang, Jiaxiong Zhu, Chunyi Zhi, Wai‐Yeung Wong

**Affiliations:** ^1^ Department of Applied Biology & Chemical Technology and Research Institute For Smart Energy The Hong Kong Polytechnic University Hong Kong P.R. China; ^2^ Department of Mechanical Engineering The University of Hong Kong Hong Kong P.R. China; ^3^ The Hong Kong Polytechnic University Shenzhen Research Institute Shenzhen P.R. China

**Keywords:** alkaline electrolytes, asymmetric metalloporphyrin, tetrahydroxozincate, zinc battery

## Abstract

Green electrochemical energy storage is essential for carbon neutrality, and alkaline zinc batteries offer a compelling solution due to their inherent safety, low cost, and high energy density. However, their performance is limited by parasitic reactions, including corrosion, gas evolution, and slow Zn/ZnO conversion kinetics stemming from inefficient dissociation of the tetrahydroxozincate [Zn(OH)_4_^2−^] intermediate. We address this by designing a series of cobalt porphyrins (Co‐4N, Co‐3N‐O, Co‐3N‐S) that modulate the metal center's charge density for accelerating Zn(OH)_4_^2^^−^ decomposition, and control Zn^2^^+^ transport through the carboxyl‐functionalized peripheries. The Co‐3N‐O‐modified electrolyte achieves exceptional stability, maintaining stable cycle for over 80,000 s at 5 mA cm^−^^2^, which is more than four times longer than the <20,000 s achieved by the conventional KOH + ZnO electrolyte. In Zn||Ni batteries, this molecularly engineered electrolyte enables 110 stable cycles at 1 mA cm^−2^, significantly outperforming the unmodified system, which sustained only 20 cycles. These findings elucidate a structure‐kinetics relationship for zincate regulation and demonstrate how customized molecular asymmetry can overcome persistent challenges in aqueous battery chemistry, offering a pathway to high‐performance, durable energy storage systems.

## Graphical abstract

This research tackles the limitations of alkaline zinc batteries by designing a series of metalloporphyrin molecules (Co‐4N, Co‐3N‐O, Co‐3N‐S) as electrolyte additives. The asymmetric structures, particularly Co‐3N‐O, accelerate the Zn(OH)_4_^2^^−^ decomposition via a modulated metal center and control Zn^2^^+^ transport through carboxyl groups. This approach enables the stable cycles of 80,000 s at 5 mA cm^−2^. It demonstrates a molecular solution to challenges in aqueous battery chemistry.

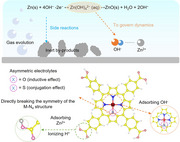

## 1 Introduction

**FIGURE 1:**
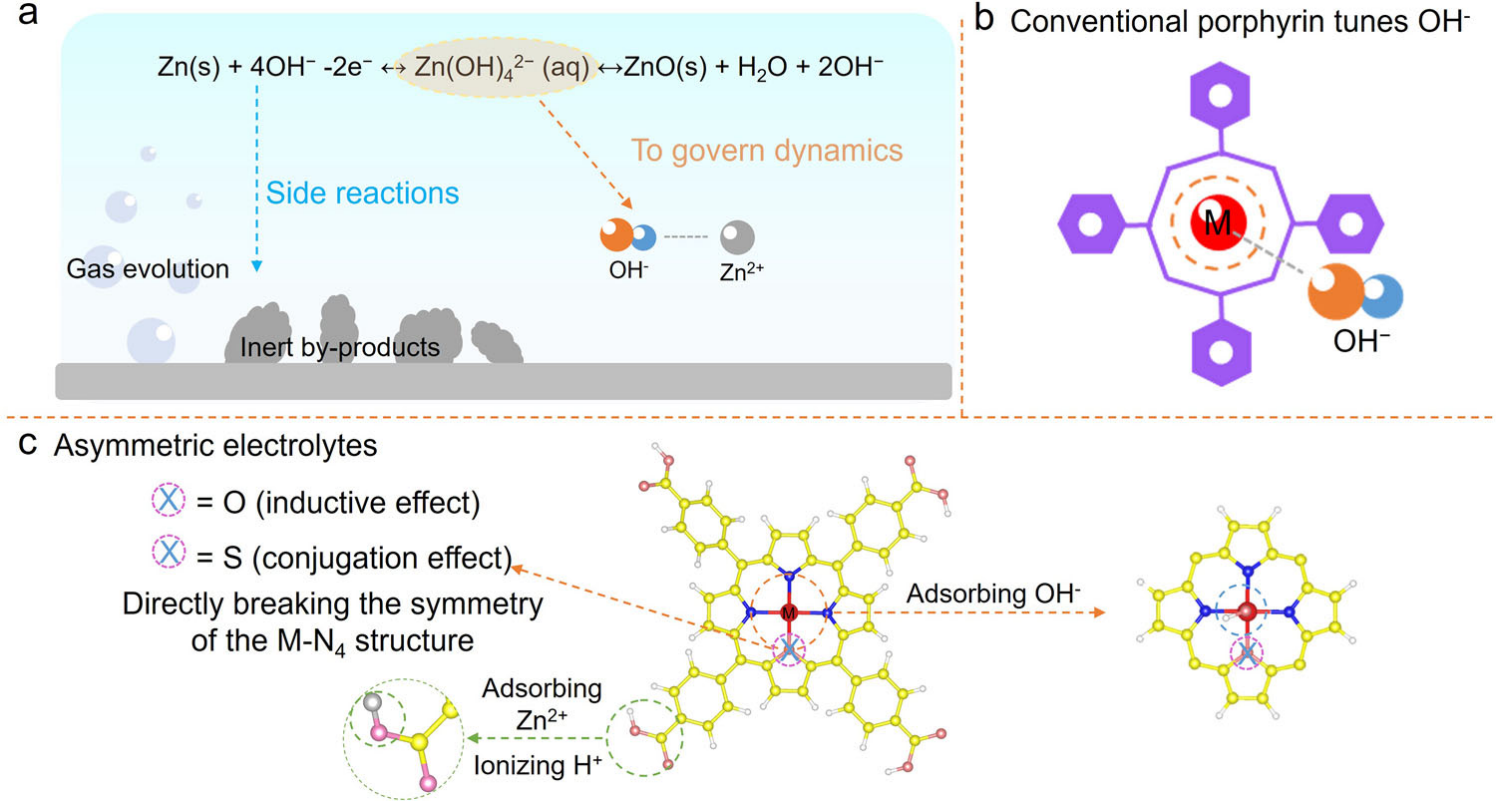
Schematic diagram of (a) the challenges faced by alkaline batteries, (b) conventional porphyrin structures for tuning OH^−^, and (c) optimization of the reaction kinetics of alkaline zinc batteries by asymmetric porphyrin‐structured electrolyte additives in this work.

The imperative need for green electrochemical energy storage emerges as the pivotal pathway toward achieving carbon neutrality [1, 2, 3, 4]. Alkaline zinc batteries, distinguished by high safety, low cost, substantial volumetric capacity, and high energy density, stand out as promising contenders for large‐scale energy storage applications [5, 6, 7]. However, alkaline electrolytes still suffer from issues such as severe corrosion, gas evolution, undesirable reaction dynamics, and electrode passivation, which lead to premature short‐circuiting and low utilization of electrode materials (Figure 1a) [8, 9]. The sluggish conversion reaction between Zn and ZnO and the formation of an inert by‐product are one of the determining factors affecting kinetics of response, which ultimately manipulate battery life and energy output [10, 11]. Current strategies to enhance redox reaction behaviors focus on mitigating side reactions and by‐product passivation, for example, by utilizing porous anode structure [12, 13], optimizing reaction path [14, 15, 16, 17], and developing optimized anode interfaces [18, 19, 20]. However, these strategies overlook an important intermediate tetrahydroxozincate [Zn(OH)_4_^2−^], which is considered the main way for Zn and ZnO to convert. Therefore, maximizing the formation and dissociation rate of Zn(OH)_4_^2−^ is essential for accelerating the Zn/ZnO conversion reaction.

Electrolyte engineering via molecular or coordination compounds is a widely used strategy for controlling reaction pathways in zinc‐based batteries [21, 22, 23]. These additives function primarily by modulating the behavior of Zn(OH)_4_^2^^−^, through mechanisms such as direct coordination to influence deposition process kinetics [24], selective adsorption to suppress deleterious anode deformation [25], and the redistribution of zincate to achieve spatially uniform plating [26]. In principle, Zn(OH)_4_^2−^ possesses a central zinc atom in the +2 oxidation state coordinated with four hydroxide (OH^−^) groups [27, 28]. Rapid removal of OH^−^ ligands from Zn(OH)_4_^2^^−^ may substantially accelerate Zn^2^^+^ deposition rate. Inspired by this, a rationally designed electrolyte that could rapidly remove hydroxide groups from Zn(OH)_4_^2−^ is expected to improve the Zn/ZnO conversion efficiency. Porphyrin molecules offer outstanding benefits, including atomic adjustability and structural stability, making them promising candidates for facilitating battery redox reactions (Figure 1b) [29, 30, 31]. The metal center constitutes the principal catalytic site in porphyrin‐mediated electrochemical processes, with its redox behavior critically dependent on the coordination geometry [32, 33]. Typically, metalloporphyrins predominantly exhibit a symmetric M‐N_4_ configuration, and this structural paradigm imposes electronic constraints that fundamentally limit redox versatility. Deliberating ligand‐field asymmetry, when combined with controlled charge density modulation at the metal center, can establish tunable OH^−^ coordination dynamics. Therefore, the assumption of substituting an N atom in the porphyrin ring with an O atom (inductive effect) and an S atom (conjugative effect) is proposed to modulate the charge density of metal center, leading to Zn(OH)_4_^2^^−^ dissociation dynamics regulation (Figure 1c). Additionally, the introduced carboxyl group is expected to ionize in alkaline electrolytes, forming negatively charged species that coordinate with Zn^2^^+^ [21, 22, 23], thereby suppressing OH^−^ coordination with Zn^2+^ and enhancing the Zn/ZnO conversion efficiency.

**FIGURE 2:**
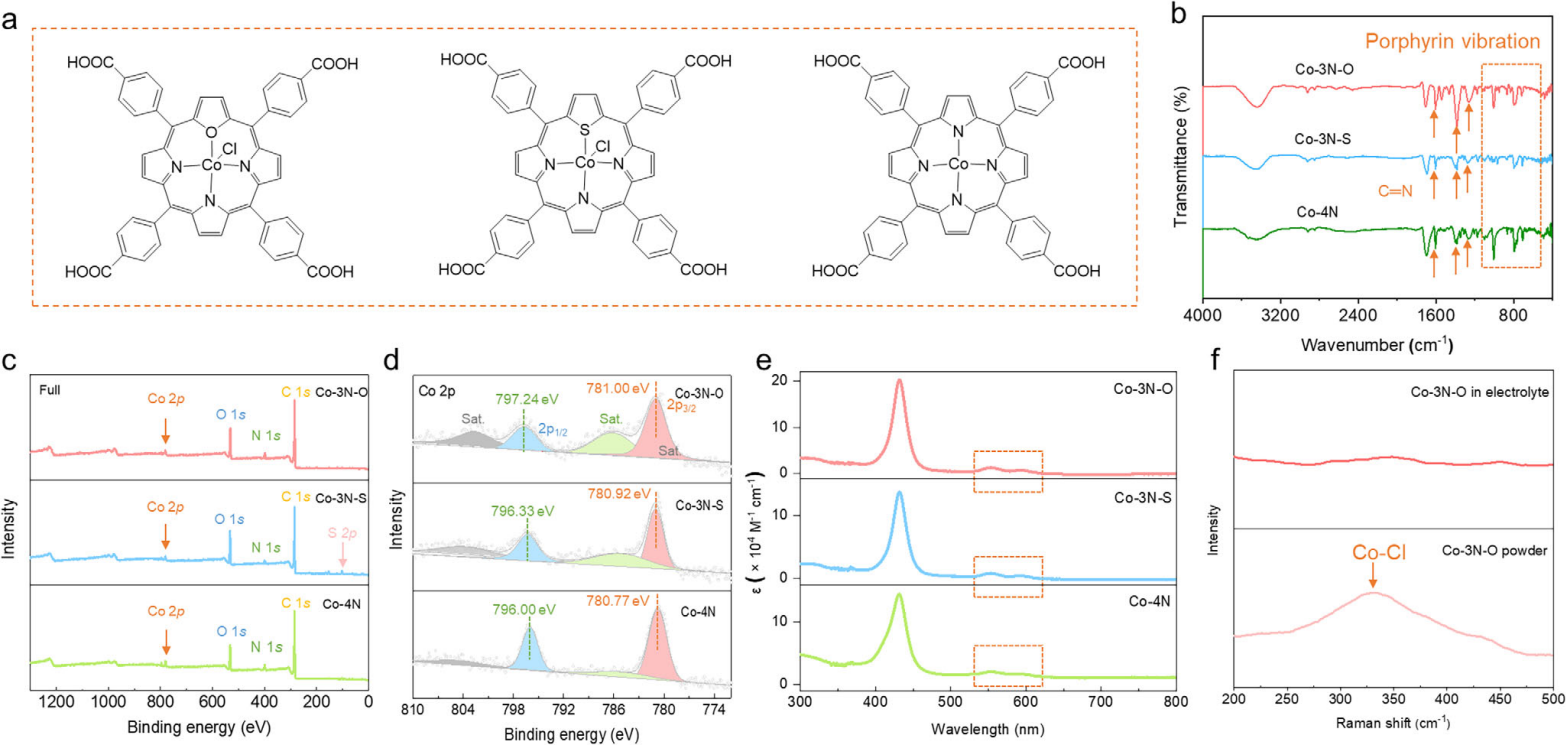
(a) Chemical structures of three porphyrins: Co‐3N‐O, Co‐3N‐S, and Co‐4N. (b) FTIR spectra of Co‐3N‐O, Co‐3N‐S, and Co‐4N. (c) Full XPS spectra and (d) Co *2p* fittings of Co‐3N‐O, Co‐3N‐S, and Co‐4N. (e) UV‐vis spectra of Co‐3N‐O, Co‐3N‐S, and Co‐4N. (f) Raman spectra of Co‐3N‐O in electrolyte and powder state.

Based on the above conjectures, we designed and synthesized a series of asymmetrized/symmetrized metalloporphyrin‐based molecules featuring oxygen (O), sulfur (S), and nitrogen (N) atoms as electrolyte additives (denoted as Co‐3N‐O, Co‐3N‐S, and Co‐4N, respectively) (Figure 2a). The performance‐enhancing effect of these additives stems primarily from their ability to accelerate reaction kinetics and improve long‐term cycling stability. Specifically, the highly electronegative O atom in the porphyrin framework coordinates with the cobalt center, promoting favorable interaction with OH^−^. This interaction facilitates rapid dissociation of Zn(OH)_4_^2^^−^, thereby accelerating the conversion reaction. Concurrently, the carboxyl groups ionize in the alkaline electrolyte, generating COO^−^ anions that coordinate with Zn^2^^+^, thus disrupting the Zn^2^^+^‐OH^−^ coordination structure. As a result, Co‐3N‐O based electrolyte presents exceptional cycling stability across different current densities, with around 80,000 s at 1 mA cm^−2^ and 1 mAh cm^−2^, 90,000 s at 5 mA cm^−2^ and 1 mAh cm^−2^ and 45,000 s at 10 mA cm^−2^ and 1 mAh cm^−2^. In contrast, conventional electrolytes (KOH + ZnO) deteriorate quickly, with 20,000 s, 16,000 s, and 6000 s at the corresponding currents of 1, 5, and 10 mA cm^−2^, respectively. Pairing a nickel cathode with the Co‐3N‐O–based electrolyte substantially enhances the long‐term electrochemical performance of Zn–Ni batteries. At the current density of 5 mA cm^−^^2^, Co‐3N‐O delivered an areal capacity of 0.83 mAh cm^−^^2^ and 92.4% Coulombic efficiency (CE) over 57 stable cycles, which outperforms the KOH + ZnO electrolyte, with 0.25 mAh cm^−^^2^ and 91.7% CE over only 13 cycles. Even at packed configuration (electrode area: 3 × 3 cm), Co‐3N‐O sustained a high specific capacity of 1.85 mAh cm^−2^ and 92.3% CE over 110 cycles, outperforming conventional electrolytes that failed after 26 cycles (1.70 mAh cm^−^^2^, 84.8% CE) due to severe anode corrosion, which highlights the promise of asymmetric metalloporphyrin molecular design for practical battery applications.

## 2 Results and Discussion

### 2.1 Synthesis and Characterization of Metalloporphyrin Molecules

The synthesis of O‐ and S‐substituted asymmetric metalloporphyrins (Co‐3N‐X, where X represents O or S) alongside the symmetric metalloporphyrin counterpart (Co‐4N) was accomplished by utilizing the previously established methodologies, with minor modifications implemented [34]. In summary, methyl 4‐formylbenzoate was dissolved in propionic acid containing pyrrole and subsequently refluxed under a nitrogen atmosphere for 12 h. This was followed by a series of washings, yielding tetramethyl 4,4′,4′′,4′′′‐(porphyrin‐5,10,15,20‐tetrayl)tetrabenzoate (4N‐Por). The next step involved the addition of cobalt chloride to 4N‐Por in *N,N*‐dimethylformamide (DMF), where the mixture was refluxed at 150°C for 6 h, again followed by requisite washings to generate Co‐4N‐Por. For the synthesis of Co‐3N‐X‐Por (X = O/S), dimethyl 4,4'‐(furan/thiophene‐2,5‐diylbis(hydroxymethylene))dibenzoate (DCPX) was prepared from methyl‐4‐formylbenzoate and furan/thiophene. Subsequently, a mixture of DCPX, methyl‐4‐formylbenzoate, and pyrrole was stirred in anhydrous dichloromethane (CH_2_Cl_2_) at room temperature in the dark for 2 h; the addition of 2,3‐dichloro‐5,6‐dicyano‐1,4‐benzoquinone (DDQ) facilitated the final formation of 3N‐X‐Por. The synthetic procedure employed for Co‐3N‐X‐Por (Co‐3N‐O‐Por and Co‐3N‐S‐Por) mirrored that utilized for Co‐4N‐Por. It involved the addition of cobalt chloride, which was subjected to reflux in DMF at 150°C for 6 h, followed by a series of requisite washings. The final products, Co‐4N and Co‐3N‐X, were synthesized through hydrolysis of Co‐4N‐Por and Co‐3N‐X‐Por with potassium hydroxide (KOH) in tetrahydrofuran (THF) at 70°C, followed by pH adjustment using hydrochloric acid (HCl). The comprehensive details of these procedures are provided in the Supporting Information.

The chemical structures of Co‐4N and Co‐3N‐X were characterized by Fourier‐transform infrared spectroscopy (FTIR) and X‐ray photoelectron spectroscopy (XPS). FTIR spectra show characteristic porphyrin‐stretching vibrations in the range of 600 to 1000 cm^−1^, with additional peaks at around 1300, 1400, and 1600 cm^−1^ corresponding to porphyrinic C─N bonds, confirming the presence of porphyrin units in all samples (Figure 2b) [35, 36]. Analyses of full XPS spectra of Co‐4N and Co‐3N‐O revealed prominent peaks corresponding to the Co, O, N, and C core elements, while Co‐3N‐S additionally showed the S signal (Figure 2c) [36, 37, 38]. In the Co *2p* region, Co‐4N exhibited two distinct peaks at 780.77 and 796.00 eV, corresponding to 2*p*_2/3_ and 2*p*_1/2_ states, respectively, characteristic of Co^II^ ion. Comparatively, Co‐3N‐O and Co‐3N‐S displayed binding energy shifts to higher values (781.00/797.24 eV and 780.92/796.33 eV, respectively). These observed blue shifts are hypothesized to be caused by the replacement of anionic N donors with neutral O and S, resulting in a decrease in electron density around Co center (Figure 2d), which is expected to facilitate OH^−^ adsorption. UV‐vis spectral analysis of metalloporphyrin was then conducted to exhibit the distinctive absorption features of Co‐3N‐X and Co‐4N (Figure 2e). The Q bands, appearing as wide peaks between 500 and 700 nm, are attributed to the a_2u_(π)→e_g_^*^(π) transition. The observed splitting of the Q band in Co‐3N‐X versus Co‐4N reflects their lower symmetry [39, 40]. The chemical structure alteration of metalloporphyrin when exposed to electrolyte was further investigated through Raman spectroscopy. The results exhibit a distinctive Co─Cl bond shift at approximately 300 cm^−1^ in solid Co‐3N‐X, yet disappearing in the electrolyte, which indicates Co─Cl bond ionization upon dissolution (Figures 2f and S1). Additionally, pH measurements showed that additives of Co‐4N and Co‐3N‐X slightly reduced the alkalinity of KOH + ZnO electrolyte, suggesting no increased corrosiveness (Figure S2).

### 2.2 Rapid Conversion Reaction Kinetics Enabled by the Porphyrin Metal Center

**FIGURE 3:**
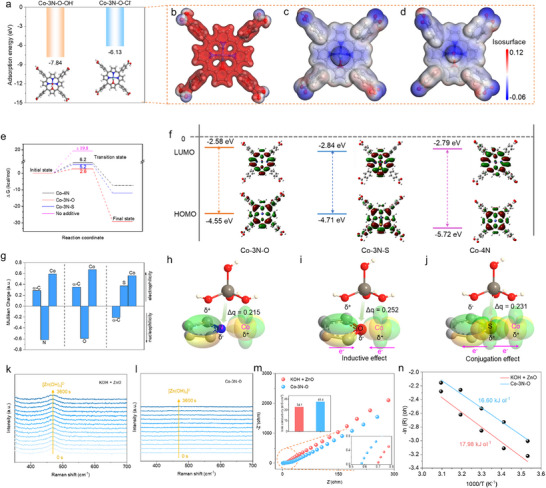
(a) Calculated adsorption energy of Co‐3N‐O for OH^−^ and Cl^−^, and the corresponding ESP maps of (b) pure Co‐3N‐O, (c) Co‐3N‐O with Cl^−,^ and (d) Co‐3N‐O with OH^−^. (e) Gibbs energy changes of Zn(OH)_4_^2−^ from adsorption to dissociation. (f) HOMO and LUMO levels of Co‐3N‐O, Co‐3N‐S, and Co‐4N. Schematic diagram of charge distribution upon binding of (h) pyrrole‐Co, (i) furan‐Co, and (j) thiophene‐Co with Zn(OH)_4_^2−^, and (g) the corresponding Mulliken charge. In situ Raman spectrum of (k) KOH + ZnO and (l) Co‐3N‐O electrolyte under a cathodic current of 10 mA from 0 to 3600 s. (m) EIS of KOH + ZnO and Co‐3N‐O for the calculation of ionic conductivities. (n) EIS at various temperatures of KOH + ZnO and Co‐3N‐O for calculating activation energies (*E*_a_).

To elucidate the enhanced conversion reaction kinetics enabled by the porphyrin metal center, a combination of theoretical simulations and experimental tracking was conducted. In alkaline zinc batteries, reversible energy storage occurs through the ZnO/Zn redox process, which proceeds via two steps: (i) ZnO + H_2_O + 2OH^─^ → Zn(OH)_4_^2─^, followed by (ii) Zn(OH)_4_^2─^ + 2e^─^ → Zn + 4OH^─^, where Zn(OH)_4_^2─^ serves as the key intermediate [41, 42]. Accelerating the dissociation of Zn(OH)_4_^2─^ is crucial for enhancing the Zn/ZnO conversion kinetics. This can be achieved through two mechanisms: (i) disruption of Zn^2^^+^‐OH^─^ coordination bonds via carboxyl groups, and (ii) strong OH^─^ adsorption at the metal center of metalloporphyrin. First, it is necessary to determine the state of porphyrin molecules in the electrolyte. As revealed by the Raman spectra of aqueous asymmetric porphyrins, axial Cl^─^ binds with Co^2+^ in an ionic bond state, which separates from the Co center before contacting with OH^─^. Density functional theory (DFT) calculations confirmed this behavior, showing a stronger adsorption energy of Co‐3N‐O for OH^−^ (−7.84 eV) than for Cl^−^ (−6.13 eV), and demonstrating its OH^−^ selective binding in electrolytes (Figure 3a). To delve deeper into the adsorption capacity of OH^−^ on the porphyrin center, the electrostatic potential (ESP) maps of Co‐3N‐O, Co‐3N‐O without Cl^−^, and Co‐3N‐O with OH^−^ were analyzed (Figure 3b–d). The absence of Cl^−^ of Co‐3N‐O reveals pronounced electrophilicity characteristics in the porphyrin center. Similar analyses for Co‐3N‐S and Co‐4N were also evaluated, yielding an important insight into their differing affinities for Cl^−^ and OH^−^ (Figure S3). Specifically, the adsorption energies of Co‐3N‐S for Cl^−^ and OH^−^ were measured at −5.57 eV and −7.19 eV, respectively, with the corresponding ESP profiles reflecting a similar trend to that of Co‐3N‐O. Co‐4N exhibited a weaker adsorption energy for OH^−^ (−4.48 eV), attributed to the absence of Cl^−^ induced electrophilicity enhancement.

Next, the coordination pathway of OH^−^ and COO^−^ was analyzed, showing stronger adsorption of Co‐3N‐O‐COO^−^ to Zn^2+^ (−4.13 eV) compared with OH–Zn^2+^ coordination (−3.79 eV), which reveals that the coordination structure of Zn(OH)_4_^2−^ would be suppressed energetically and replaced by metalloporphyrin structure (Figure S4a). Moreover, the adsorption energies of Zn^2+^ to Co‐3N‐S‐COO^−^ and Co‐4N‐COO^−^ were measured as −4.04 eV and −6.46 eV, demonstrating the high nucleophilicity of both 3N‐S‐COO^−^ and 4N‐COO^−^ to Zn^2+^ ions (Figure S4b). Furthermore, the reaction pathway of Zn(OH)_4_^2−^ dissociation was simulated. As portrayed in Figure 3e, during the dissociation of Zn(OH)_4_^2−^, the breaking of Zn−O bonds is extremely difficult (calculated dissociation energy is over 19.8 kcal/mol via relaxed scan method). When Co‐3N‐X (X = N, O, S) additives are introduced, the energy barrier (transition state) required for dissociation decreases sharply, indicating that the addition of these additives significantly facilitates the dissociation of Zn(OH)_4_^2−^. Furthermore, compared to Co‐4N and Co‐3N‐S, Co‐3N‐O exhibits the lowest dissociation energy barrier for Zn(OH)_4_^2−^ (2.6 kcal/mol), indicating that Co‐3N‐O is more conducive to Zn(OH)_4_^2−^ dissociation. This finding is consistent with its superior battery cycling performance. For the detailed energy changes and computational structures, we can refer to Figure S5. To fundamentally unveil the reason behind the energy barrier of Zn(OH)_4_^2−^ dissociation via Co‐3N‐X (X = N, O, S) additives, we systematically calculated and analyzed the altered electrophilicity of the Co center induced by O/S substitution. For the frontier orbital (HOMO‐LUMO) distribution (Figure 3f), when pyrrole N in porphyrin is substituted by O, the O atom's additional lone pair electrons delocalize within the porphyrin ring, thus elevating the HOMO energy level [43, 44, 45, 46, 47]. This narrows the HOMO‐LUMO energy gap, thereby enhancing charge transport properties. When pyrrole N is substituted with S, not only do the S lone pair electrons elevate the HOMO, while the unique empty d orbital of S also lowers the LUMO energy level [48], further enhancing the charge transport properties of Co‐3N‐S. However, the charge transfer capability (HOMO‐LUMO) of the additive is not the key factor determining the dissociation of Zn(OH)_4_^2−^. The critical factor lies in the inductive/conjugation effects of O/S on Co and Zn. For the pyrrole‐Co structure (Co‐4N moiety), the electronegativity of N induces positive charge on the pyrrole α‐carbon and Co (Figure 3h). Consequently, Co and α‐C jointly bind to OH^−^, while Zn binds to N, collectively promoting the dissociation of Zn(OH)_4_^2−^ (Figure 3g). For the furan‐Co structure (Co‐3N‐O fragment), the higher electronegativity of O increases the positive charge on Co and α‐C (larger Mulliken charge), enhancing the polarization strength between Zn and OH^−^ and further favoring the dissociation of Zn(OH)_4_^2−^ (Figure 3i). In the thiophene‐Co structure (Co‐3N‐S fragment), the empty d orbitals of S participate in porphyrin delocalization (Figure 3j) [48].

The *d* orbitals of S overlap with those of Zn, and the resulting orbital conjugation effect also contributes to the dissociation of Zn(OH)_4_^2−^. From a charge transfer perspective, the charge transfer for Zn(OH)_4_^2−^ bound to Co‐4N is 0.215, to Co‐3N‐O is 0.252, and to Co‐3N‐S is 0.231. These results indicate that both the inductive effect of furan O and the conjugation effect of thiophene S promote the dissociation of Zn(OH)_4_^2−^, with the inductive effect of furan O being more significant. This O/S electronic analysis aligns with the aforementioned energy barrier analysis of the transition states for Zn(OH)_4_^2−^ dissociation (Figure 3e), providing a charge and orbital perspective on the effect of additives Co‐3N‐X (X = N, O, S) on Zn(OH)_4_^2−^ dissociation. These findings underscore the distinct adsorption capability of metal center in porphyrin for OH^−^ and shed light on the intricate dynamics of molecular adsorption processes.

Following a theoretical analysis of OH^−^ adsorption on metalloporphyrin, in situ Raman spectroscopy was employed to track the reaction intermediates. Under an operating current of 10 mA, the characteristic Zn(OH)_4_^2^^−^ peak (∼468 cm^−^^1^) remained clearly detectable for 3600 s in the KOH + ZnO electrolyte (Figures 3k,l and S6). In contrast, this signal was negligible in the Co‐3N‐O system, indicating that the metalloporphyrin electrolyte alters the reaction pathway, by suppressing the accumulation of Zn(OH)_4_^2^^−^ intermediate and accelerating the conversion reaction rate for Zn. Moreover, a quantitative assessment of the kinetic parameters of the Zn anode experiments was conducted. The ionic conductivity of Co‐3N‐O, as determined from electrochemical impedance spectroscopy (EIS), was found to be 41.4 mS cm^−2^, surpassing that of the conventional electrolyte, which was measured at 34.1 mS cm^−2^ (Figure 3m). The enhanced conductivity is primarily ascribed to the Co‐3N‐O, which facilitates the dissociation of Zn(OH)_4_^2−^ ions. This process increases the available charge carrier density, leading to improved conductivity. Similarly, the ionic conductivities of Co‐4N and Co‐3N‐S were measured to be 41.5 and 34.9 mS cm^−2^, respectively, both of which exceeded that of the conventional electrolyte (Figure S7). The surface wetting properties of different electrolytes on the electrode surface were assessed throughout 0 s to 15 s, demonstrating improved wettability for Co‐3N‐O, Co‐3N‐S, and Co‐4N, which indicates lower diffusion resistance (Figures S8 and S9). To further illustrate the circumstances of the reaction conversion process, the activation energies (*E*_a_) were calculated based on EIS at various temperatures, revealing values of 16.60 and 17.98 kJ mol^−1^ for Co‐3N‐O and the conventional electrolyte, respectively (Figures 3n and S10). The zinc conversion reaction requires overcoming the activation energy barrier, wherein a lower activation energy facilitates a more facile conversion process. Additionally, the open circuit potential over 3000 s under constant current conditions was analyzed, presenting a lower potential for Co‐3N‐O compared to the conventional electrolyte (Figure S11). According to Ohm's law, the lower potential indicates reduced reaction resistance. Under constant potential, the current density was monitored over 400 s. Co‐3N‐O exhibited a higher current density than the conventional electrolyte, further demonstrating its lower reaction resistance (Figure S12).

### 2.3 Performance Evaluation of the Long‐Life Zinc Anode

**FIGURE 4:**
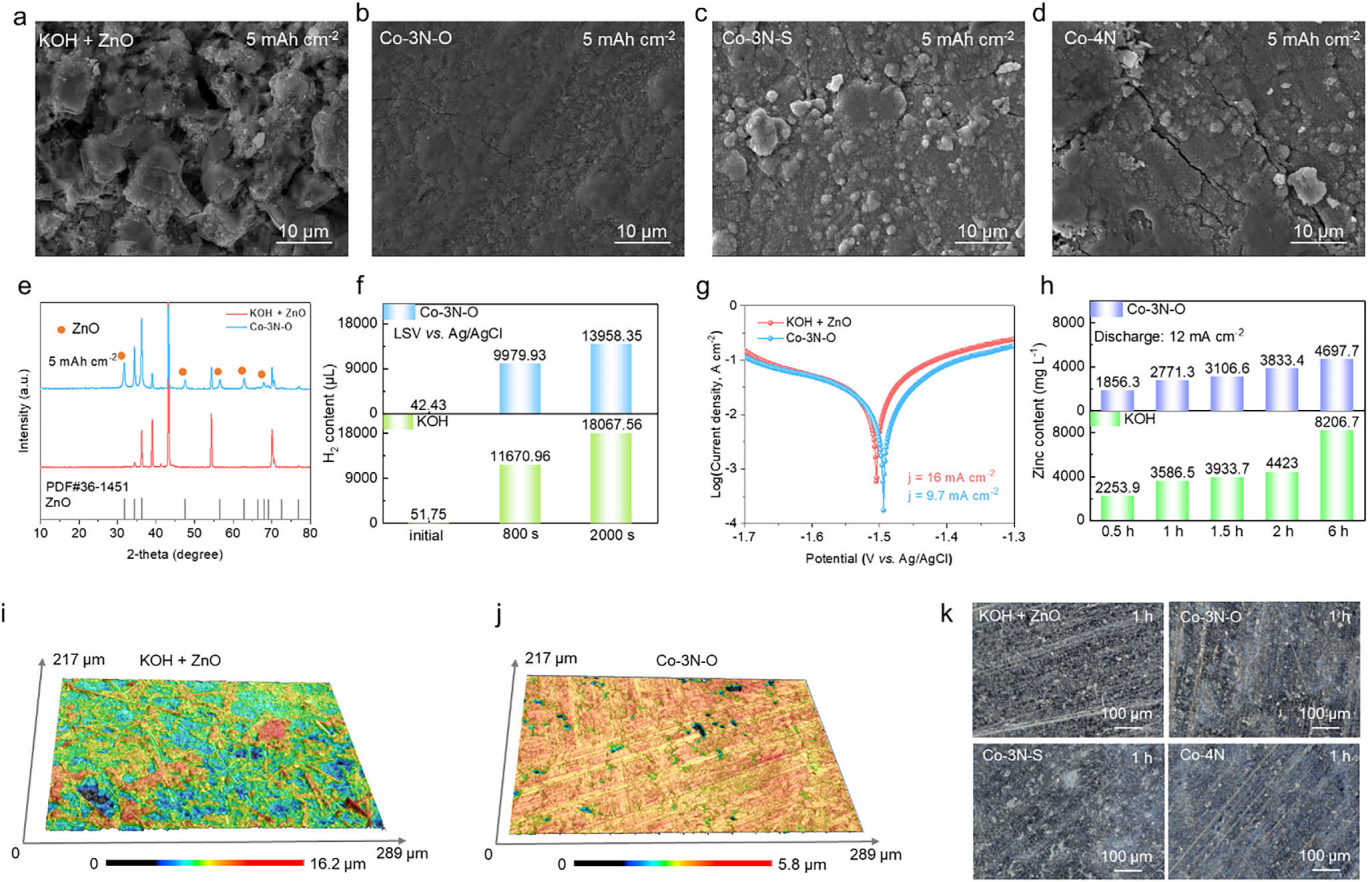
Zinc anode surface morphology of (a) KOH + ZnO, (b) Co‐3N‐O, (c) Co‐3N‐S, and (d) Co‐4N at 5 mAh cm^−2^. (e) XRD spectra of the zinc anode of KOH + ZnO and Co‐3N‐O at 5 mAh cm^−2^. (f) The hydrogen content produced after LSV testing in the potential range of −0.1 to −0.2 V vs Ag/AgCl was analyzed by gas chromatography (GC). (g) Tafel curves of KOH + ZnO and Co‐3N‐O electrolyte systems. (h) Zinc content of Co‐3N‐O and KOH electrolyte after discharging at 12 mA cm^−2^ from 0.5 to 6 h. (i) CLSM 3D height images of zinc anode after immersing for 48 h in (i) KOH + ZnO and (j) Co‐3N‐O electrolytes. (k) Zinc anode surface morphology after immersing for 1 h in KOH + ZnO, Co‐3N‐O, Co‐3N‐S, and Co‐4N electrolytes.

Building on the enhanced conversion kinetics enabled by asymmetric porphyrins, we systematically assessed battery stability through coupled electrochemical and morphological characterization. Galvanostatic cycling at 5 mA cm^−^^2^ revealed stark contrasts in anode degradation: conventional KOH + ZnO electrolyte produced progressive corrosion features (Figure 4a), whereas the Co‐3N‐O‐modified system maintained stable electrode architectures after 1‐h operation (Figure 4b). Comparative analysis showed that Co‐3N‐S and Co‐4N additives likewise improved anode morphological stability relative to the unmodified electrolytes (Figure 4c,d). The original zinc foil surface is present for comparison (Figure S13). Additionally, X‐ray diffraction (XRD) pattern of the anode under a discharge condition of 5 mAh cm^−2^ provides a useful mechanistic insight, with clear ZnO diffraction signatures in the porphyrin‐modified systems, indicating an efficient conversion reaction from Zn to ZnO. The absence of these features in the control electrolytes further corroborated the critical role of molecular engineering in promoting the complete redox transformations (Figures 4e and S14).

A series of electrochemical characterizations was then employed to reveal the distinct interfacial stabilization by the porphyrin‐modified electrolytes. The hydrogen evolution potential was assessed through linear sweep voltammetry (LSV), presenting values of −1.96 V and −1.82 V under −30 mA cm^−2^ for Co‐3N‐O and KOH + ZnO electrolytes, respectively (Figure S15). A more negative potential indicates the increased difficulty in hydrogen evolution, suggesting a more stable interface. To intuitively compare gas evolution between the KOH and the porphyrin‐based (Co‐3N‐O) systems, hydrogen production was quantified by gas chromatography (GC) during the LSV measurement (Figure 4f). From 0 to 800 s (corresponding to −1.0 to −1.4 V vs. Ag/AgCl), where zinc oxidation/corrosion is dominant, the KOH system generated more H_2_ (11,670.96 µL) than the Co‐3N‐O system (9,979.93 µL). From 800 to 2000 s (corresponding to −1.4 to −2.0 V), where the reduction reaction and the hydrogen evolution reaction (HER) prevail, the KOH system still produced a higher H_2_ volume. This confirms that gas evolution is more severe in the KOH electrolyte. Additionally, the corrosion‐inhibiting effect of the Co‐3N‐O electrolyte was quantified using two complementary methods. First, Tafel analysis yielded a lower corrosion current density (9.7 mA cm^−^^2^) for the Co‐3N‐O system than for the conventional KOH + ZnO electrolyte (16 mA cm^−^^2^) (Figure 4g). Then, direct measurement of zinc dissolution via inductively coupled plasma optical emission spectroscopy (ICP‐OES) during discharge at 12 mA cm^−^^2^ provided further evidence. Although dissolved zinc content increased with time (0.5–6 h) in both electrolytes, the Co‐3N‐O system demonstrated consistently lower concentrations and a slower accumulation rate (Figure 4h). Also, the zinc dissolution content in the electrolyte was measured during cycling at 12 mA cm^−^^2^ and 1 mAh cm^−^^2^. The Co‐3N‐O system consistently exhibited a lower zinc concentration than the KOH system, both at 20 min and 2 h (Figure S16). Together, these data robustly confirm the protective function of the porphyrin‐based electrolyte in stabilizing the zinc anode.

The effectiveness of the modified additives in preventing anode corrosion was further confirmed by immersion tests (Figure S17). Zinc foils experienced an obvious weight loss in KOH + ZnO electrolyte after 18 and 48 h, but remained stable in the modified electrolytes. This demonstrates that the additives shield the zinc foil from a corrosive alkaline environment during idle periods. XRD patterns of post‐immersion anodes confirmed the absence of significant by‐products in all of the electrolytes (Figure S18). Laser microscope analysis of zinc anodes immersed for 18 h showcased heightened surface irregularities in the conventional electrolyte, with a fluctuation level of 16.2 µm, as opposed to only 5.8 µm in the Co‐3N‐O system (Figure 4i,j). A similar trend was observed for Co‐3N‐S and Co‐4N, accentuating their superior attributes in comparison to the conventional electrolyte (Figure S19). A more intuitive anode surface morphology was observed in Figures 4k and S20, at the immersion times of 1 and 5 h. The anode surface of porphyrin‐modified electrolyte is much flatter compared with that of the conventional electrolytes. These results demonstrate that molecularly tailored electrolytes simultaneously mitigate the electrochemical corrosion and morphological degradation through interfacial stabilization.

### 2.4 Electrochemical Performance

**FIGURE 5:**
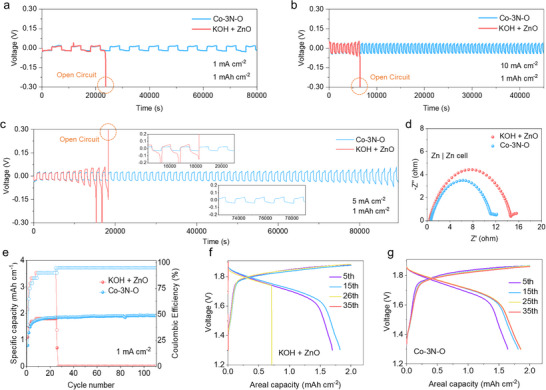
Cycling performance of KOH + ZnO and Co‐3N‐O symmetric battery at (a) 1 mA cm^−2^, (b) 10 mA cm^−2^, (c) 5 mA cm^−2^. (d) EIS curves of KOH + ZnO and Co‐3N‐O symmetric battery. (e) Cycling performance of zinc‐nickel batteries at a packed configuration (electrode area: 3 × 3 cm) and the corresponding voltage‐capacity profiles of (f) KOH + ZnO and (g) Co‐3N‐O at the current density of 1 mA cm^−2^.

The implementation of metalloporphyrin‐modified electrolyte systems yielded outstanding cycling stability across different current densities compared to the conventional electrolyte, benefiting from the advantages of accelerated reaction kinetics and stable electrolyte properties. The introduction of Co‐3N‐O resulted in a stable cycle of 80,000 s at 1 mA cm^−2^ and 1 mAh cm^−2^, while the conventional electrolyte exhibited a rapid degradation after approximately 20,000 s (Figure 5a). Noticeably, at 5 mA cm^−2^ and 1 mAh cm^−2^, Co‐3N‐O exhibited a cycle stability surpassing 90,000 s, a significant improvement compared to the conventional electrolyte (Figure 5c). Even at a higher current density of 10 mA cm^−2^ and 1 mAh cm^−2^, Co‐3N‐O still could maintain a stable cycle exceeding 45000 s, but just 6000 s for the conventional electrolyte, highlighting the effectiveness of the molecular additive in mitigating corrosion issues caused by alkaline electrolytes (Figure 5b). Long cycle tests of Co‐3N‐S and Co‐4N at 1, 5, and 10 mA cm^−2^ were also performed, and the results show better cycle stabilities compared with the conventional electrolyte (Figures S21–S23). EIS curve analysis revealed a lower impedance for Co‐3N‐O, further supporting its enhanced stability and promoting the reaction kinetics (Figure 5d). Moreover, the Co‐3N‐O metalloporphyrin electrolyte significantly enhanced the stability of Zn||Ni full battery. Co‐3N‐O delivered an areal capacity of 0.83 mAh cm^−^^2^ and 92.4% CE over 57 stable cycles, significantly outperforming the conventional electrolyte (0.25 mAh cm^−^^2^, 91.7% CE over only 13 cycles) under a high current density of 5 mA cm^−^^2^ (Figure S24). The enhanced stability is corroborated by post‐cycling scanning electronic microscopy (SEM). Anodes disassembled from the Co‐3N‐O system (5 mA cm^−^^2^, 20 cycles) displayed a dense and uniform surface. In contrast, anodes cycled with the conventional KOH+ZnO electrolyte showed severe, inhomogeneous corrosion, rationalizing its rapid failure (Figure S25). Even at a packed configuration (electrode area: 3 × 3 cm), Co‐3N‐O sustained a high specific capacity of 1.85 mAh cm^−^^2^ over 110 cycles with 92.3% CE at 1 mA cm^−^^2^, outperforming conventional electrolytes that failed after 26 cycles (1.70 mAh cm^−^^2^, 84.8% CE) due to severe anode corrosion (Figure 5e). The voltage‐capacity profiles corroborated the superior high‐voltage performance of Co‐3N‐O (Figure 5f,g). EIS curve analysis revealed a lower impedance for the zinc‐nickel battery with Co‐3N‐O, further supporting its enhanced rate capability (Figure S26). The Co‐3N‐S and Co‐4N variants also showed improvement, delivering 1.83 mAh cm^−^^2^ and 91.7% CE for 62 cycles and 1.77 mAh cm^−^^2^ and 88.7% CE for 38 cycles, respectively, both exceeding conventional system performance (Figure S27).

## 3 Conclusion

In conclusion, we proposed an asymmetrized molecular design strategy for cobalt porphyrins to govern Zn(OH)_4_^2^^−^ dynamics and interfacial stability in alkaline zinc batteries. By creating a series of asymmetrized/symmetrized cobalt porphyrins (Co‐3N‐X, X = O, S and Co‐4N), we introduce precise charge density modulation at the catalytic metal center. This tailored electronic structure, governed by inductive (O) or conjugative (S) ligand effects, promotes the selective adsorption and rapid dissociation of OH^−^ ligands from the Zn(OH)_4_^2^^−^ intermediate, thereby accelerating the critical Zn/ZnO conversion kinetics. Concurrently, the peripheral carboxylate (COO^−^) groups regulate Zn^2^^+^ transport, synergistically enhancing the overall reaction pathway. This dual‐function electrolyte additive, particularly Co‐3N‐O, delivers exceptional interfacial stability, suppressing hydrogen evolution and mitigating zinc corrosion. As a result, symmetric Zn||Zn cells achieve remarkable cycling longevity—exceeding 90,000 s at 5 mA cm^−^^2^—far outperforming conventional electrolytes. When integrated into Zn||Ni full batteries, the molecularly engineered electrolyte enables stable high‐capacity cycling over 110 cycles at 1 mA cm^−2^, highlighting its practical utility. Our work establishes a clear structure–kinetics relationship for governing zincate electrochemistry and illustrates how atomically precise coordination asymmetry in molecular additives can overcome fundamental bottlenecks in aqueous battery systems. This approach opens a novel pathway for advanced energy storage through the rational molecular control of reaction intermediates and interfacial processes.

## Author Contributions

The manuscript was written through contributions of all authors. All authors have given approval to the final version of the manuscript.

## Conflicts of Interest

The authors declare no conflicts of interest.

## Supporting information




**Supporting File 1**: anie71357‐sup‐0001‐SuppMat.docx.

## Data Availability

Data are available on request from the authors. The data that support the findings of this study are available from the corresponding author upon reasonable request.
