# Supplementary material for: Asymmetric Electrolytes Govern Tetrahydroxozincate Dynamics for Stable Alkaline Zinc Batteries
Source: Angew Chem Int Ed Engl. 2026 Feb 9;65(12):e24438. doi: 10.1002/anie.202524438 (PMC12990964; doi:10.1002/anie.202524438)
Supplement: Supplementary file 1 — Supporting File 1: anie71357‐sup‐0001‐SuppMat.docx. [file ANIE-65-e24438-s001.docx]

**Asymmetric Electrolytes** **Govern** **Tetrahydroxozincate Dynamics for Stable Alkaline Zinc Batteries**

Xianhong Chen^1^, Yang Wang^1,^*, Jiaxiong Zhu^2^, Chunyi Zhi^2,^* and Wai-Yeung Wong^1^^,^*

X. Chen, Y. Wang, W.-Y Wong

Department of Applied Biology & Chemical Technology and Research Institute for Smart Energy, The Hong Kong Polytechnic University, Hung Hom, Hong Kong, P.R. China

E-mail: yang1.wang@polyu.edu.hk; wai-yeung.wong@polyu.edu.hk.

X. J. Zhu, C. Zhi

Department of Mechanical Engineering,

The University of Hong Kong, Hong Kong, P.R. China

E-mail: yang1.wang@polyu.edu.hk; wai-yeung.wong@polyu.edu.hk.

**Supporting Information**

1. **Experimental section**

**Synthesis of tetramethyl 4,4’,4’’,4’’’-(porphyrin-5,10,15,20-****tetrayl)tetrabenzoate (4N-Por)**

4N-Por was synthesized through the utilization of a previously documented methodology.^[1]^ Specifically, methyl 4-formylbenzoate (6.9 g, 42 mmol) was dissolved in 100 mL of propanoic acid, followed by the incremental addition of pyrrole (3.0 mL, 43 mmol). The reaction mixture was subsequently subjected to reflux for 12 hours under constant stirring. Upon cooling to room temperature, the resultant mixture underwent filtration, followed by sequential washings with 50 mL of methanol and 50 mL of acetone. The resultant product was then dried to yield the desired compound as a purple solid form (55% yield based on methyl 4-formylbenzoate).

**Synthesis of Co-4N****-Por**

Co-4N-Por was synthesized via the metallation of 4N-Por, following the reported methods with slight modifications.^[1]^ Stoichiometric amounts of CoCl_2_·6H_2_O (119.0 mg, 0.5 mmol) and 4N-Por (423.5 mg, 0.5 mmol) were dissolved in anhydrous dimethylformamide (DMF) (20 mL) under a N_2_ atmosphere, and the mixture was refluxed at 150 ℃ for 6 h. After cooling to ambient temperature, the resulting mixture was extracted with chloroform (100 mL), washed with water (3 × 50 mL), and dried over anhydrous Na_2_SO_4_. The solvent was evaporated under reduced pressure to yield a purple solid (85.3% yield based on 4N-Por).

**Synthesis of DCPX, where X represents S or O** (dimethyl 4,4'-(furan-2,5-diylbis(hydroxymethylene))dibenzoate (DCPO), and dimethyl 4,4'-(thiophene-2,5-diylbis(hydroxymethylene))dibenzoate (DCPS))

The synthesis of DCPX was adapted from the previously reported literature with minor modifications. ^[2]^ Anhydrous hexane (30 mL) was transferred into a 250 mL two-necked round-bottom flask equipped with a rubber septum, a gas inlet, and a gas outlet tube. The flask was purged with N_2_ gas for 5 minutes before the addition of TMEDA (3.7 mL, 25 mmol) and *n*-BuLi (10.0 mL of a 2.5 M hexane solution) at room temperature, followed by the introduction of furan (1.45 mL) or thiophene (1.60 mL). The reaction mixture was refluxed at 90 °C for 1 hour. The formation of the 2,5-dilithium salt of furan or thiophene was ascertained by the appearance of a white, turbid solution as the reaction progressed. Following the completion of the reflux, the mixture was cooled in an ice bath, and methyl 4-formylbenzoate (3.6 g, 22 mmol) dissolved in anhydrous THF (60.0 mL) was added dropwise to the stirred suspension. After stirring the mixture at 0 °C for 1 h, it was allowed to reach room temperature, and the reaction was quenched by the addition of cold saturated NH_4_Cl solution (50 mL). The organic phase was washed with brine and then dried over anhydrous Na_2_SO_4_. The solvent was subsequently removed under reduced pressure using rotary evaporation, yielding a crude product that was further purified by silica gel column chromatography employing an eluent of ethyl acetate/hexane (v/v, 1:2). This process culminated in the isolation of the desired DCPX as a white solid (2.45 g, 31.0% yield based on furan, 3.50 g, 42.4% yield based on thiophene).

**Synthesis of 3N-X-Por (****3N-S-Por and 3N-O-Por)**

The synthesis of 3N-X-Por was conducted by employing suitable modifications derived from the reported literature methodologies.^[1b, 2a, 2b]^ A reaction mixture comprising DCPX (where X represents O, furan, 792.8 mg; S, thiophene, 824.9 mg), methyl 4-formylbenzoate (656.2 mg, 4.0 mmol), and pyrrole (420 μL, 6 mmol) was prepared in anhydrous CH_2_Cl_2_ (200 mL) under a nitrogen atmosphere. This mixture underwent cyclocondensation following the addition of boron trifluoride diethyl etherate (BF_3_·OEt_2_) (25 μL, 0.2 mmol). The reaction was allowed to stir at ambient temperature in the absence of light for 2 h. Subsequently, 2,3-dichloro-5,6-dicyano-1,4-benzoquinone (DDQ) (1.36 g, 6.0 mmol) was introduced, and the mixture was stirred under a nitrogen atmosphere for 1 h. The purification of the reaction products involved the removal of the solvent under reduced pressure, followed by separation of the resultant compounds through silica gel column chromatography utilizing a DCM/acetone eluent (100:1 *v/v*). This process led to the isolation of solid 3N-X-Por (3N-O-Por 254.4 mg, 15% yield based on DCPO; 3N-S-Por 214.3 mg, 12% yield based on DCPS).

**Synthetic routes of Co-3N-X-Por (Co-3N-O-Por, Co-3N-S-Por)**

The synthesis of Co-3N-X-Por was conducted by utilizing a similar methodology to that employed for Co-4N-Por.^[1a]^ Specifically, CoCl_2_·6H_2_O (119.0 mg, 0.5 mmol) and 3N-X-Por (172.8 mg for 3N-O-Por; 182.2 mg for 3N-S-Por) were dissolved in 10 mL of DMF within a nitrogen atmosphere. The resultant mixture was subjected to stirring and reflux at 150 °C for 5 h. Following a gradual cooling to room temperature, the reaction mixture was transferred into 100 mL of chloroform. The organic phase was subsequently washed with water (3 × 50 mL) and dried over anhydrous Na_2_SO_4_. Evaporation of the solvent under reduced pressure yielded a purple solid.

**Synthetic routes of** **Co-4N and Co-3N-X**

The synthesis of Co-4N (or Co-3N-X) was accomplished by reacting 0.132 mmol of Co-4N-Por (or Co-3N-X-Por) with 15 mL of a 7% aqueous KOH solution in 30 mL of tetrahydrofuran (THF), which was stirred at 70 °C overnight. Subsequently, the pH of the system was adjusted to 1-2 using 2 M HCl, resulting in the formation of a crystalline solid with a yield of 98%. Co-4N, MALDI-TOF (m/z, found 847.412, calculated 847.124, formula: [M^+^]). Co-3N-O, MALDI-TOF (m/z, found 849.194, calculated 884.085, formula: [M-Cl^-^]). Co-3N-S, MALDI-TOF (m/z, found 865.046, calculated 900.062, formula: [M-Cl^-^]).

1. **Characterizations**

Raman spectra were measured by the Renishaw Micro-Raman Spectroscopy System. X-ray diffraction (XRD) data were collected by a Rigaku SmartLab 9kW with X-ray source (λ ~ 1.54Å). Field Emission Scanning Electron Microscope images were acquired by Tescan MIRA. Mass spectrometry was performed by Bruker UltrafleXtreme MALDI-TOF/TOF MS. Confocal laser scanning microscope (CLSM) images were collected from Japan Keyence VK-X1000. X-ray photoelectron spectroscopy was tested by American Thermo Scientific ESCALAB 250Xi. Fourier transform infrared (FTIR) spectra were collected on a Thermo Scientific Nicolet iS50 Fourier Transform Infrared Spectrometer. UV-vis spectra were measured by "VARIAN" CARY 4000 UV-VIS Spectrophotometer.

1. **Electrochemical measurements**

Electrochemical measurements—including electrochemical impedance spectroscopy (EIS), cyclic voltammetry (CV), linear sweep voltammetry (LSV), Tafel analysis, open-circuit potential monitoring, and linear sweep voltammetry—were performed using a CHI 660E electrochemical workstation. ZView software was employed to analyze equivalent circuit fitting parameters. Most of the electrochemical tests utilized CR2025 coin cells with a zinc foil anode (0.08 mm thick, 12 mm diameter) and a glass fiber separator (19 mm diameter, 675 µm thickness, 2.7 µm pore size). The electrolyte consisted of 6 M KOH and 0.2 M ZnO, while the modified porphyrin-electrolyte systems consisted of 0.02 wt% Co-4N, Co-3N-X additive. For symmetric cell tests, two pure Zn foils were separated by the glass fiber membrane. For zinc-nickel asymmetric cell tests, the cathode comprised spherical nickel hydroxide (β, D50=10.5μm, Co 3.7%, Zn 3.6%) and PTFE in a mass ratio of 99:1. The slurry was evenly coated on a 2 mm thick nickel foam, dried at 60°C for 12 h, and then rolled to form an electrode with a thickness of about 0.7 mm. For the zinc-nickel pouch cell, the cathode and anode measured 3 × 3 cm. For the zinc-nickel coin cell, the electrodes were circular with a diameter of 12 mm. All galvanostatic charge-discharge cycling performance at varying current densities was performed using a Land CT2001A battery tester (Wuhan, China).

The activation energy E_a_ was calculated according to the Arrhenius **equation 1**^[3]^:

$\ln\left( {R_{ct}}^{-1} \right)=\ln A-\frac{E_{a}}{RT}$ **Equation 1**

where R_ct_, A, R, and T are the charge transfer resistance, constant factor, molar gas constant, and absolute temperature, respectively.

The ionic conductivity was tested by two stainless steel (SS) electrodes (12 mm diameter), and calculated according to the following **equation 2**^[4]^:

$\sigma=\frac{L}{R_{b}S}$ **Equation 2**

where $R_{b}$ represents the resistance based on EIS measurement, L represents the thickness of the length between two electrodes, and S is the contact area of the electrode and electrolyte.

The Tafel analysis for corrosion current density was determined using the Tafel extrapolation method. The linear portions of the anodic and cathodic branches (typically > 50 mV away from the corrosion potential, *E_corr*) were fitted to the Tafel **equation 3^[5]^**:

$\eta=a+b \log\left| j \right|$ **Equation 3**

where $\eta$ is the overpotential and b is the Tafel slope. The fitted lines were extrapolated to the corrosion potential (E_corr), and the intercept of these extrapolated lines on the current density axis provided the value of j.

1. **Calculation method**

i) For the calculation of electrostatic potential:

The structural modeling and energy calculation were performed by BIOVIA Materials Studio 2020 (MS 2020) software package. First-principles calculations were performed within the framework of density functional theory (DFT) using the Dmol3 module with the frequency calculation to obtain zero-point vibrational energy and entropy. Exchange-correlation effects were treated with the Perdew-Burke-Ernzerhof (PBE) generalized gradient approximation (GGA). A double numerical basis set with d- and p-polarization (DNP) and a 1×1×1 Monkhorst-Pack *k*-point grid was employed for Brillouin zone integration during geometry optimization and single-point energy calculations. The Zn surface model comprised a four-layer slab with a 25 Å vacuum layer to prevent periodic interactions. Structural relaxations were iterated until convergence thresholds of 0.00001 eV/atom (energy) and 0.04 eV/Å (forces) were achieved.

ii) For the adsorption of Zn(OH)_4_^2−^ and search for transition states:

Density functional theory (DFT) calculations were carried out by using the Gaussian 16 package. Molecular geometries were fully optimized at the B3LYP level. The 6-31G(d,p) basis set was used for all elements except for Co and Zn, for which the LANL2DZ basis set and pseudopotential were employed. Vibrational frequency calculations at the same level were performed to verify that each stationary point was either a minimum (no imaginary frequency) or a transition state (only one imaginary frequency). The dispersion corrections were taken into consideration with Grimme's D3 method.


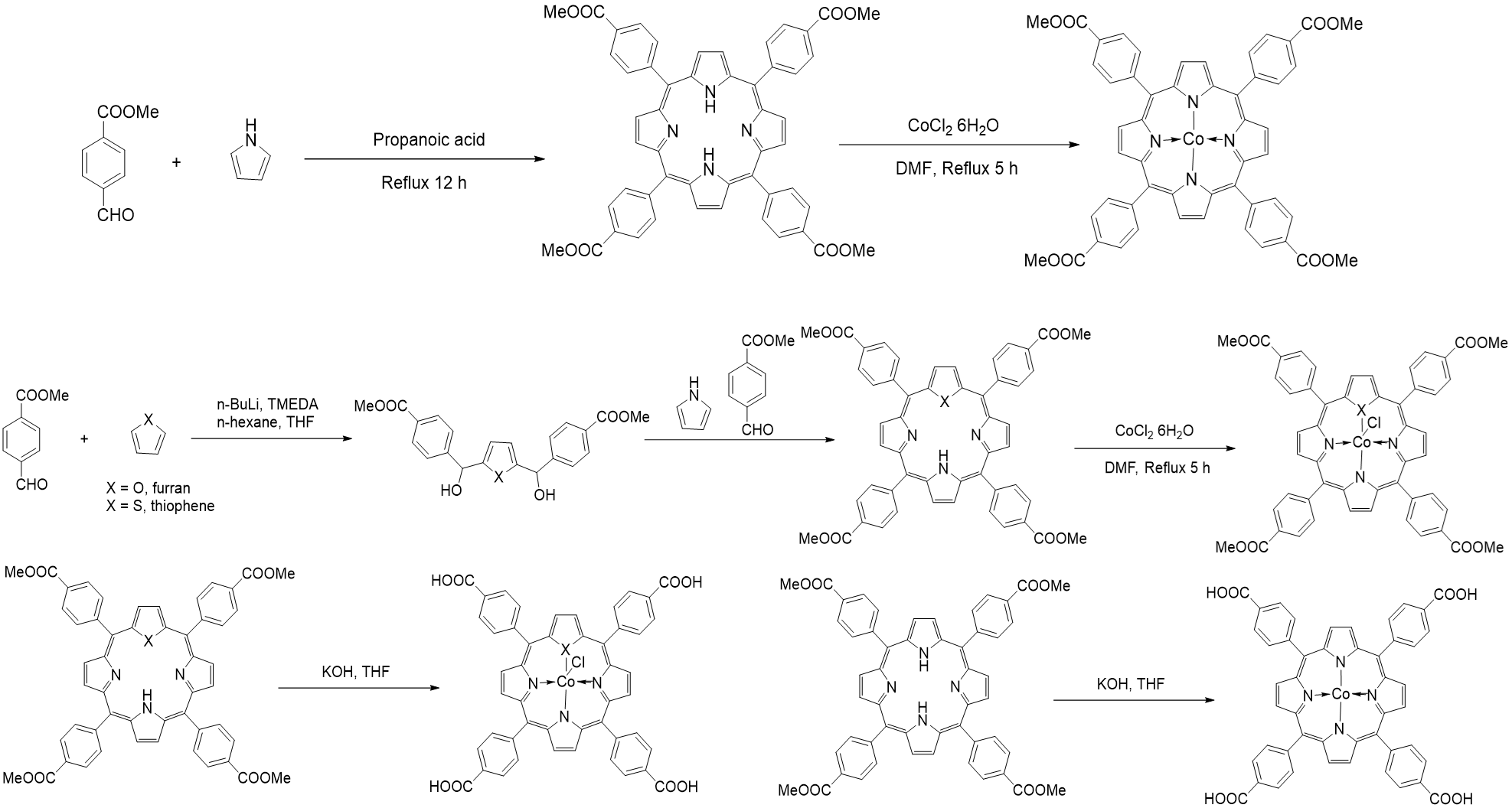


**Scheme S1**. Synthetic route of cobalt tetramethyl 4,4’,4’’,4’’’-(porphyrin-5,10,15,20-tetrayl)tetrabenzoate (Co-4N-Por) and Co-3N-X-Por (X = O/S).


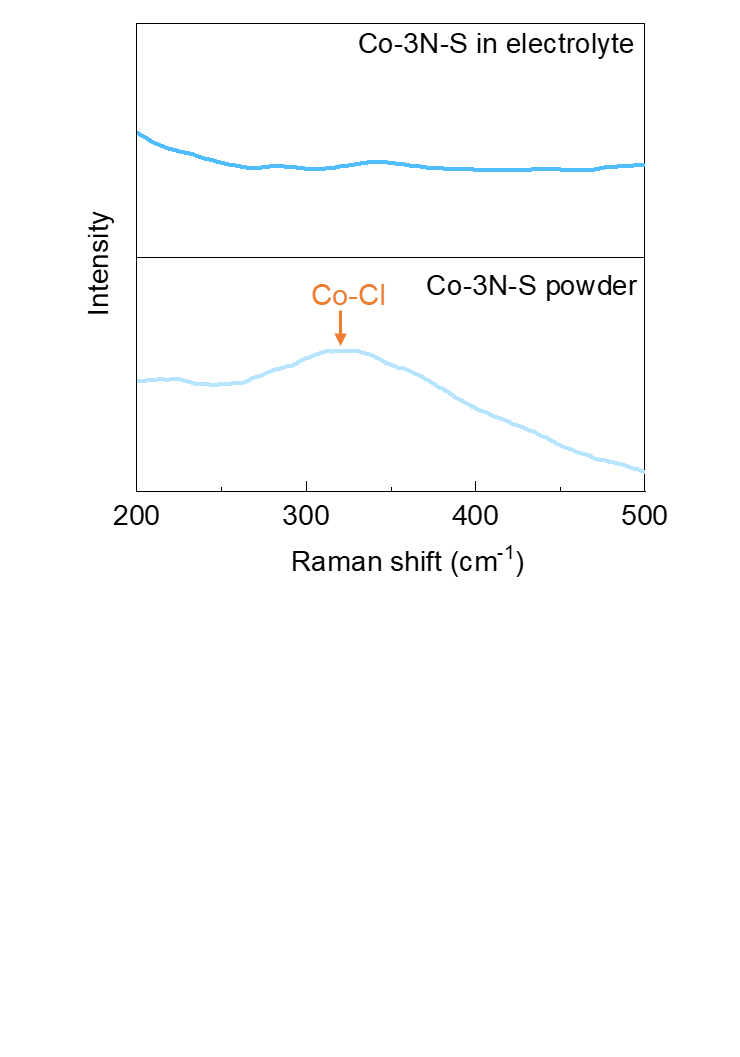


**Figure S1.** Raman spectra of Co-3N-S in electrolyte and powder state.


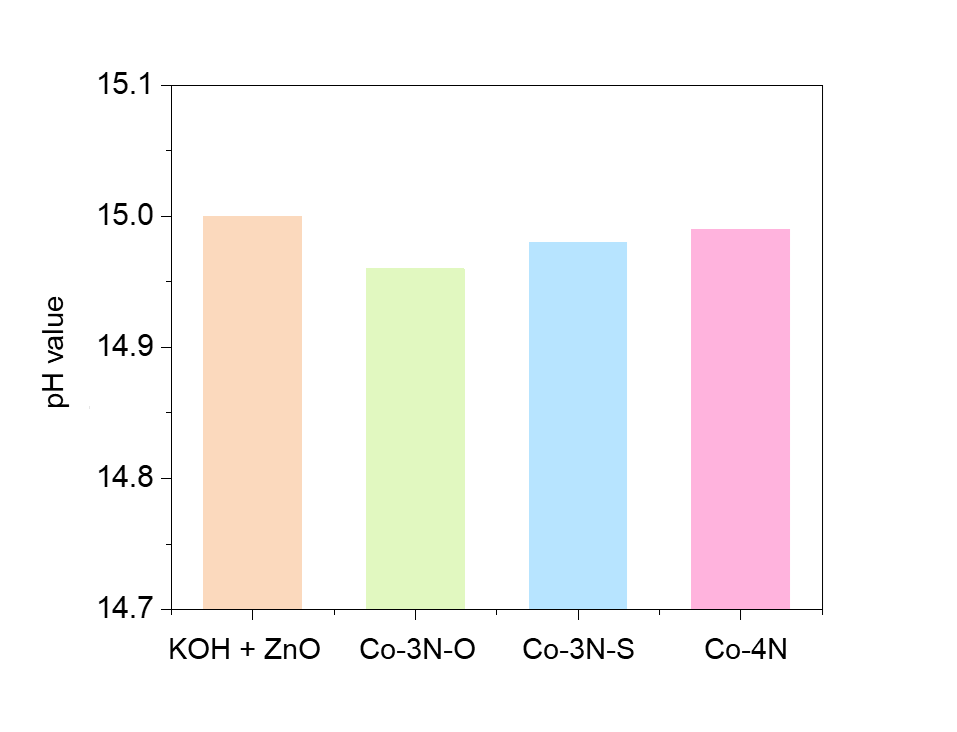


**Figure S2.** pH value of KOH + ZnO electrolyte, and electrolyte containing Co-4N and Co-3N-X additives.


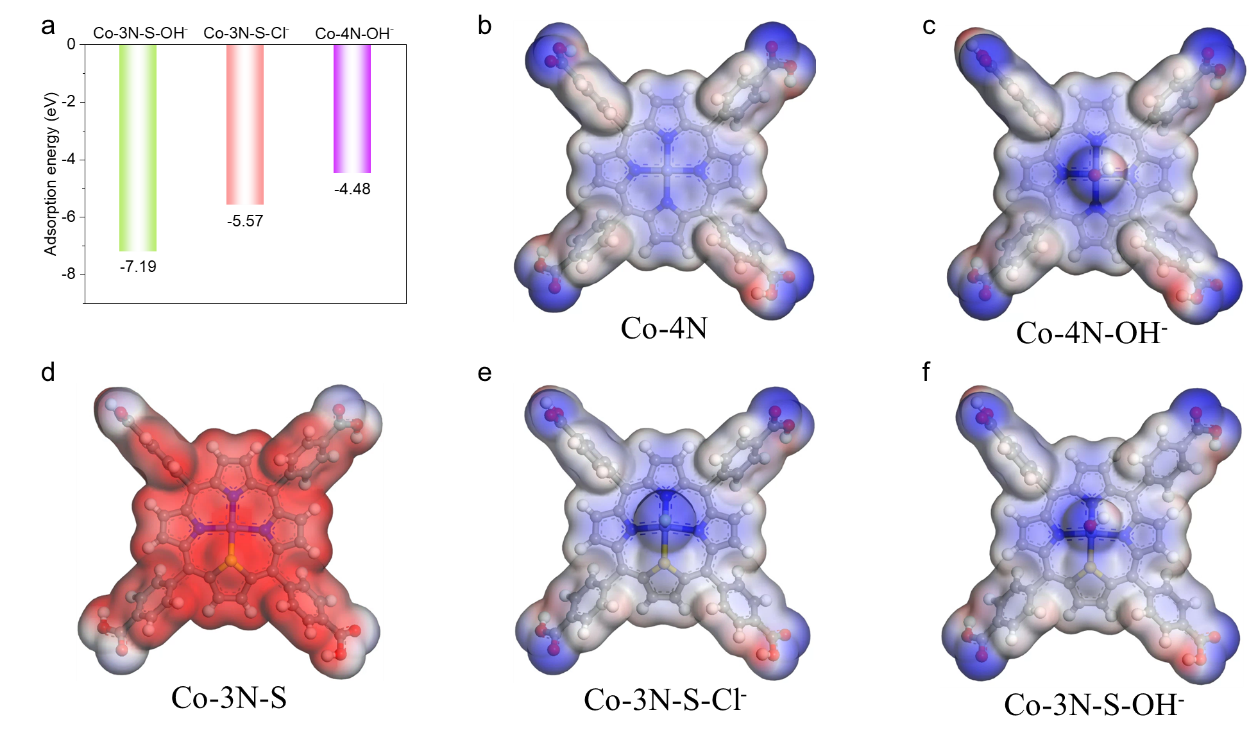
**Figure S3.** (a) Calculated adsorption energy of Co-4N for OH^-^, and Co-3N-S for OH^-^ and Cl^-^. Corresponding ESP maps of (b) Co-4N, (c) Co-4N with OH^-^, (d) pure Co-3N-S, (e) Co-3N-S with Cl^-^, and (f) Co-3N-S with OH^-^.


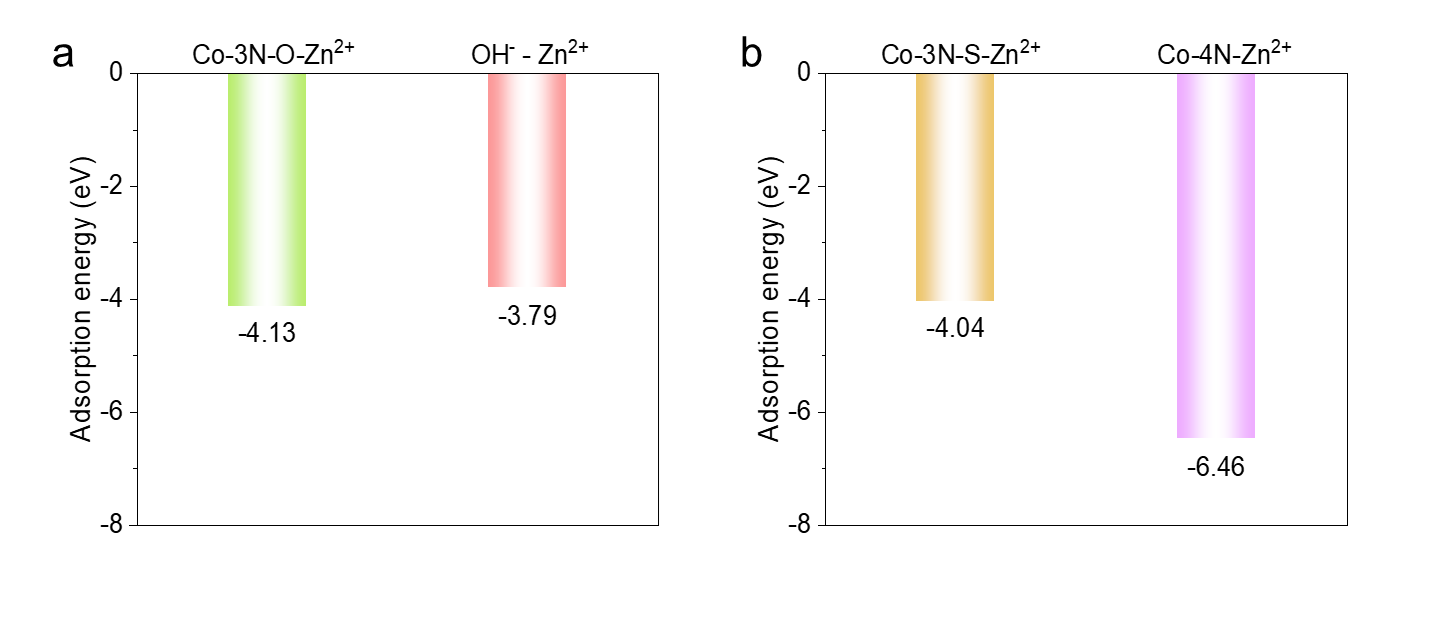


**Figure S4.** Calculated adsorption energy of (a) Co-3N-O and OH^-^ and (b) Co-3N-S and Co-4N for Zn^2+^.

**
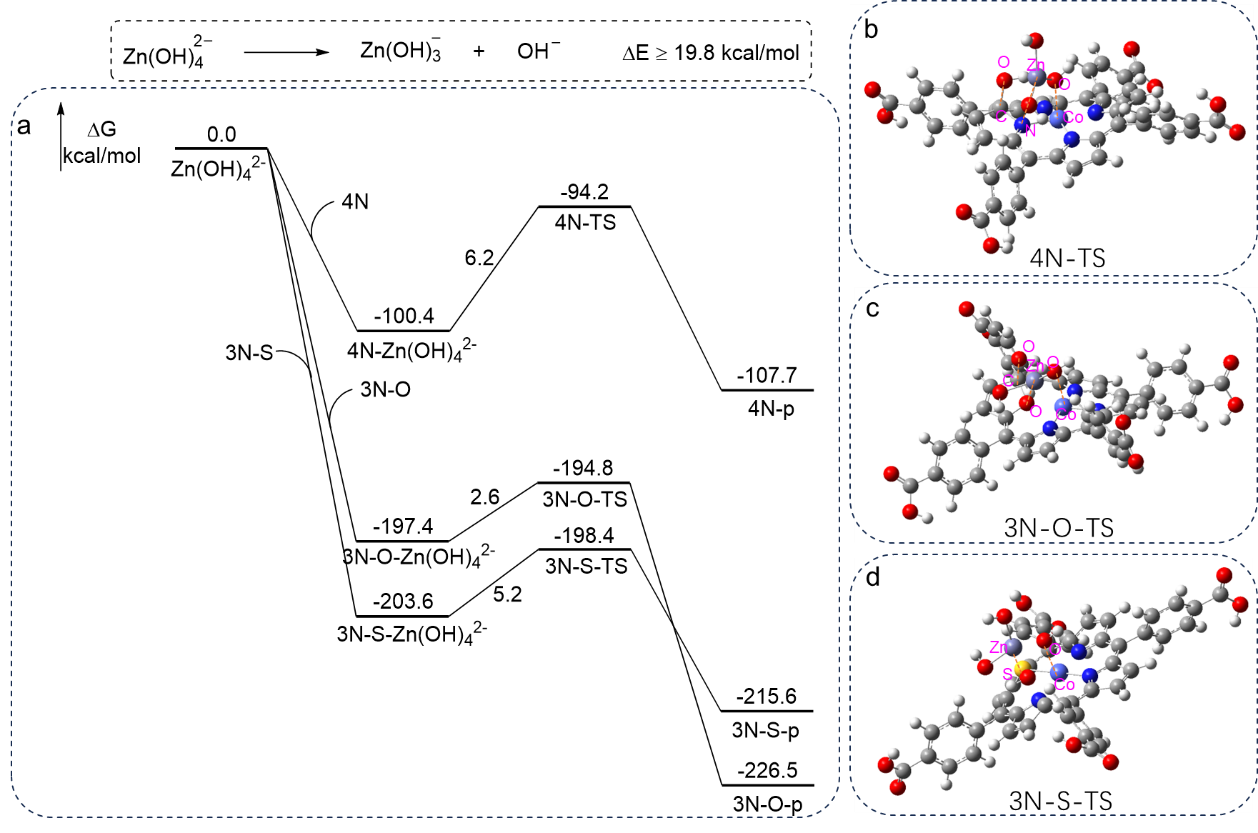
**

**Figure S5.** Detailed Gibbs energy changes of Zn(OH)_4_^2−^ and the corresponding transition state structures (b-d).

*
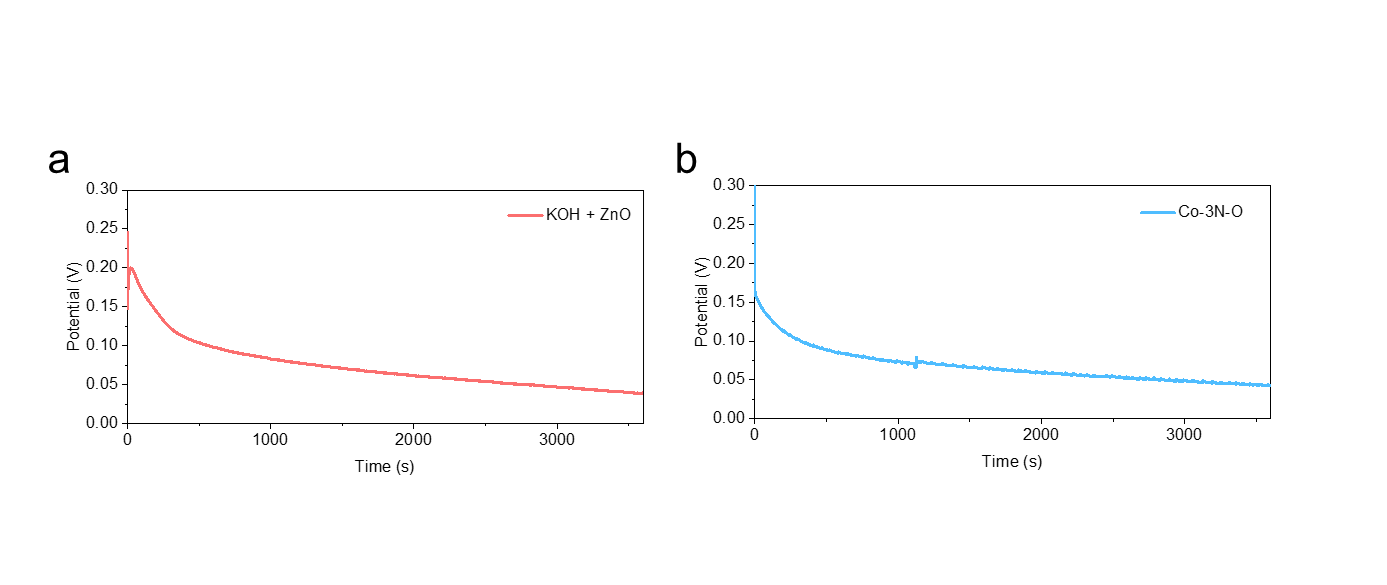
*

**Figure S6.** Chronopotentiometry (CP) curve for zinc-nickel battery with (a) KOH + ZnO and (b) Co-3N-O electrolyte under a cathodic current of 10 mA.


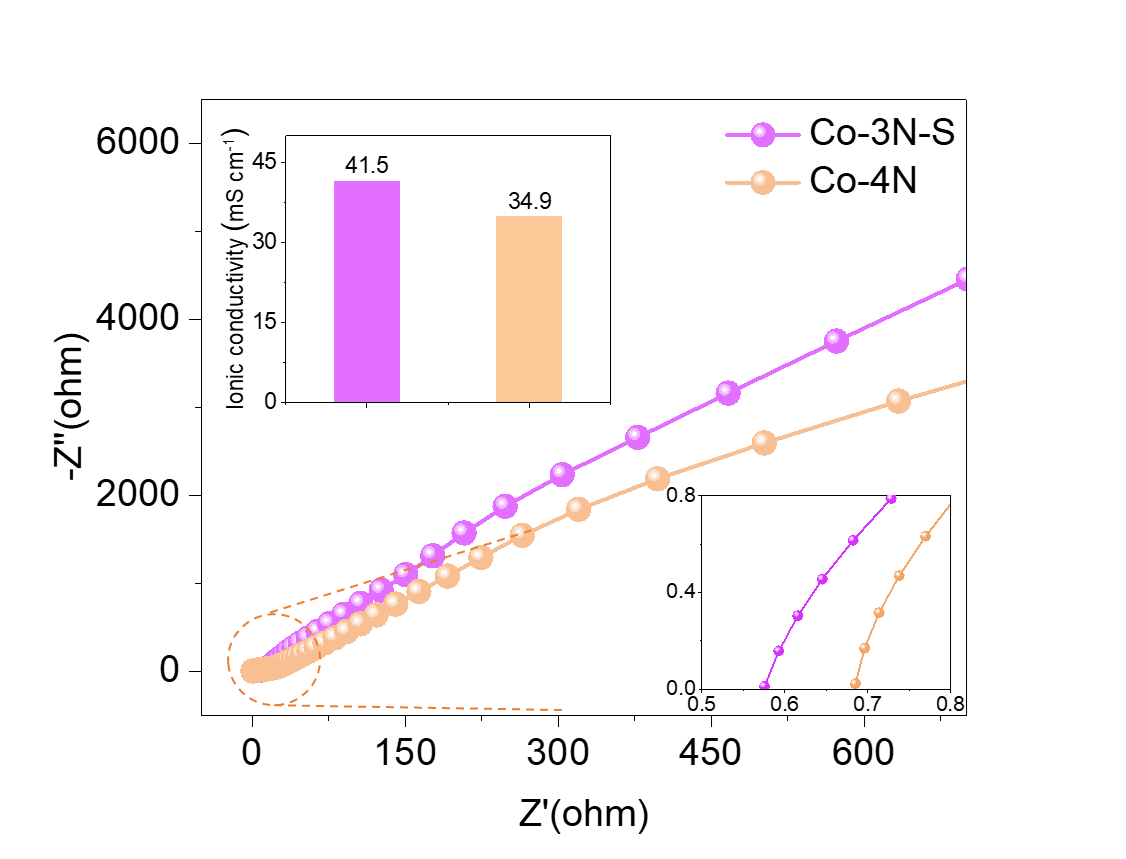


**Figure S7.** EIS maps of Co-3N-S and Co-4N for the calculation of ionic conductivities.


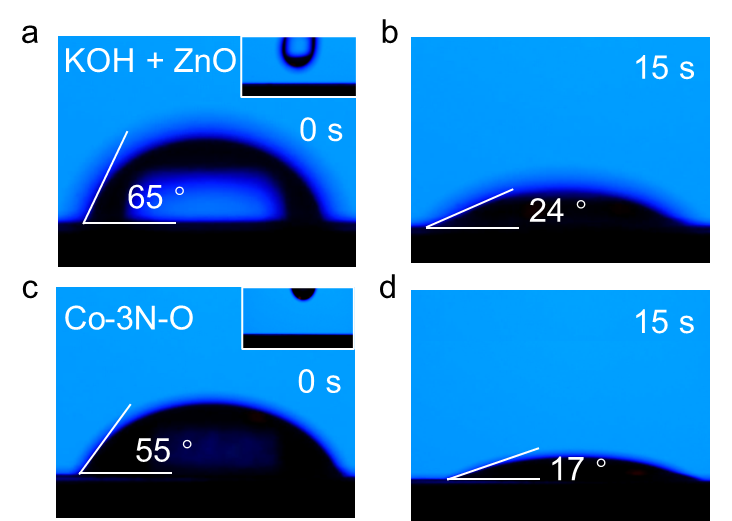


**Figure S8.** Contact angles of KOH + ZnO at (a) 0 s and (b) 15 s, and Co-3N-O at (c) 0 s and (d) 15 s on the zinc anode surface.


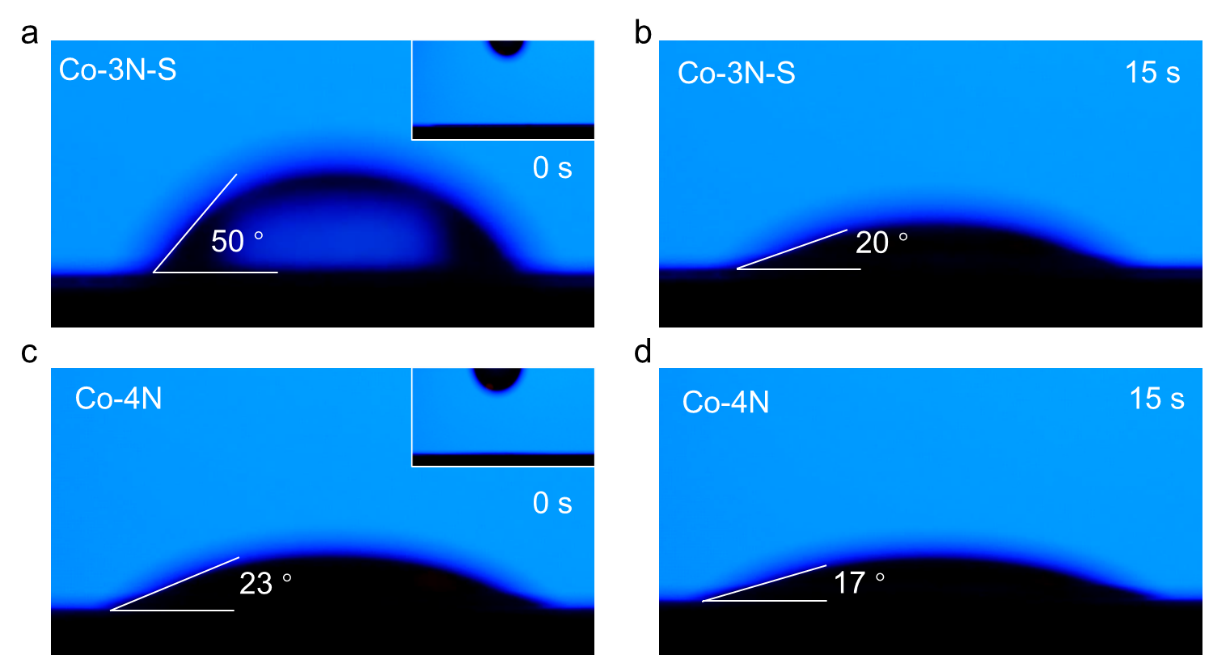


**Figure S9.** Contact angles of Co-3N-S at (a) 0 s and (b) 15 s, and Co-4N at (c) 0 s and (d) 15 s on the zinc anode surface.


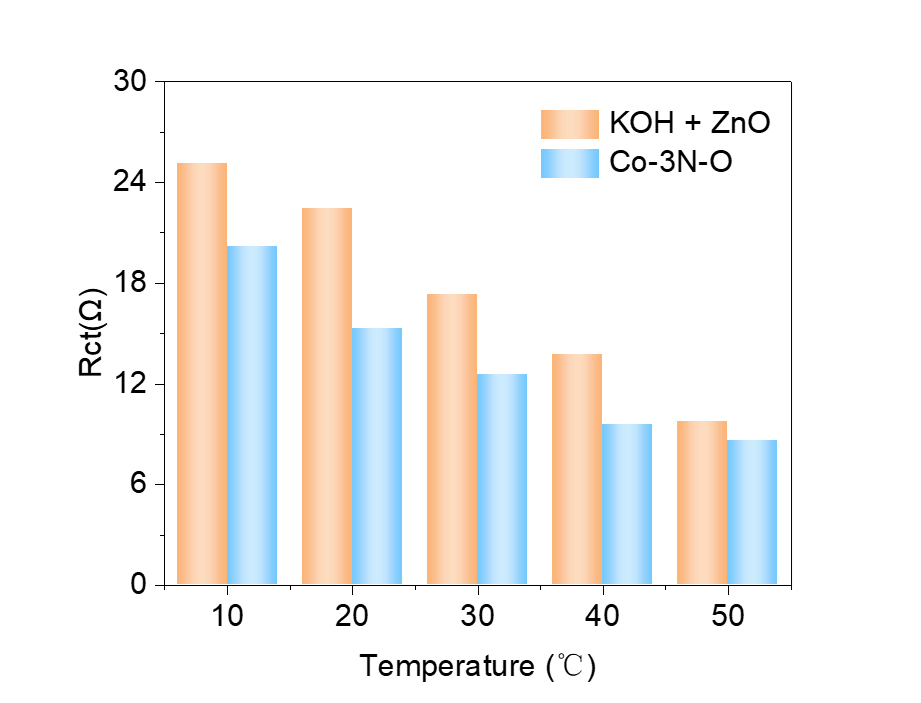


**Figure S10.** Temperature-dependent EIS spectra of (a) KOH + ZnO, b) Co-3N-O, and c) corresponding summary chart.


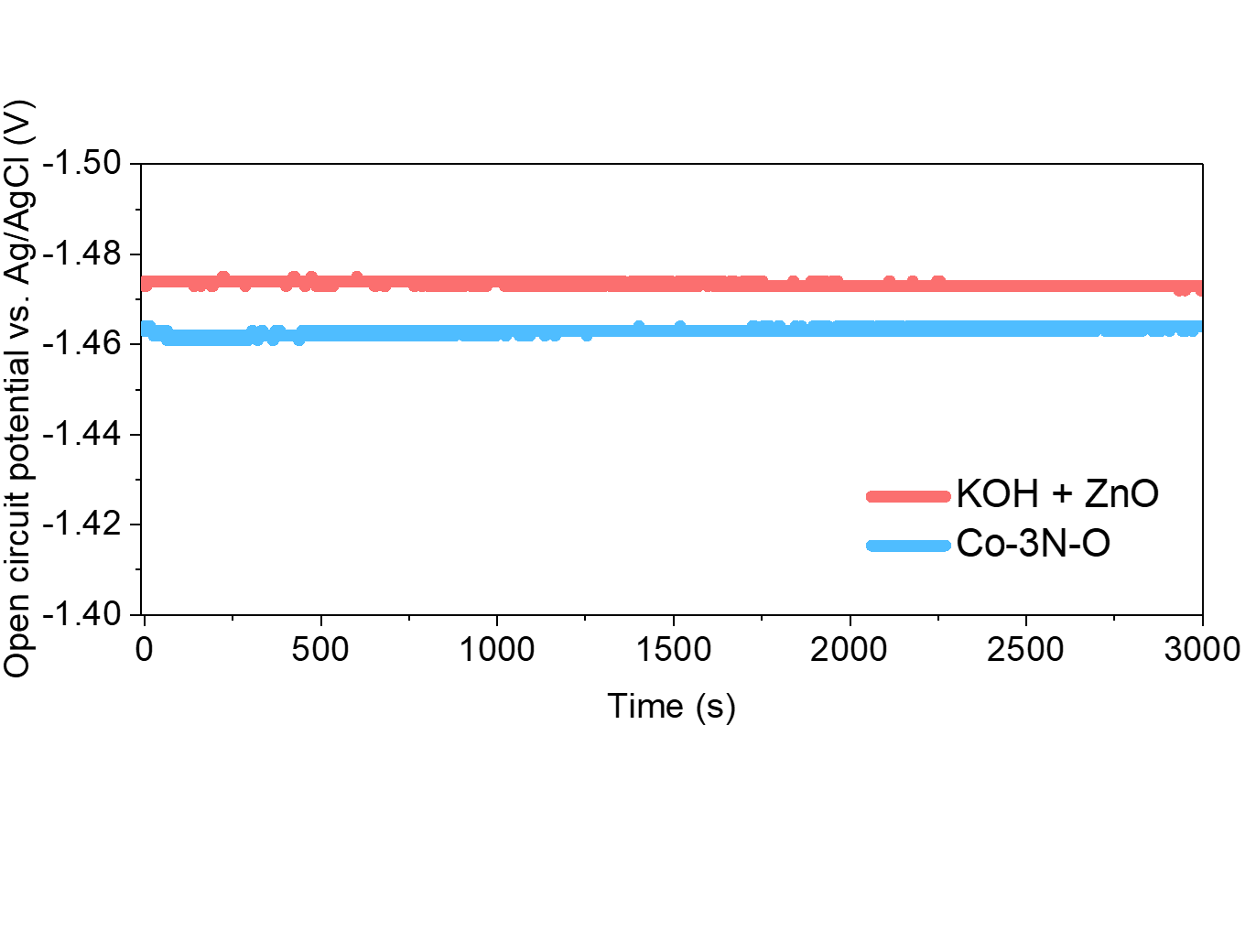


**Figure S11.** The open circuit potential of KOH + ZnO and Co-3N-O over 3000 s under constant current conditions.


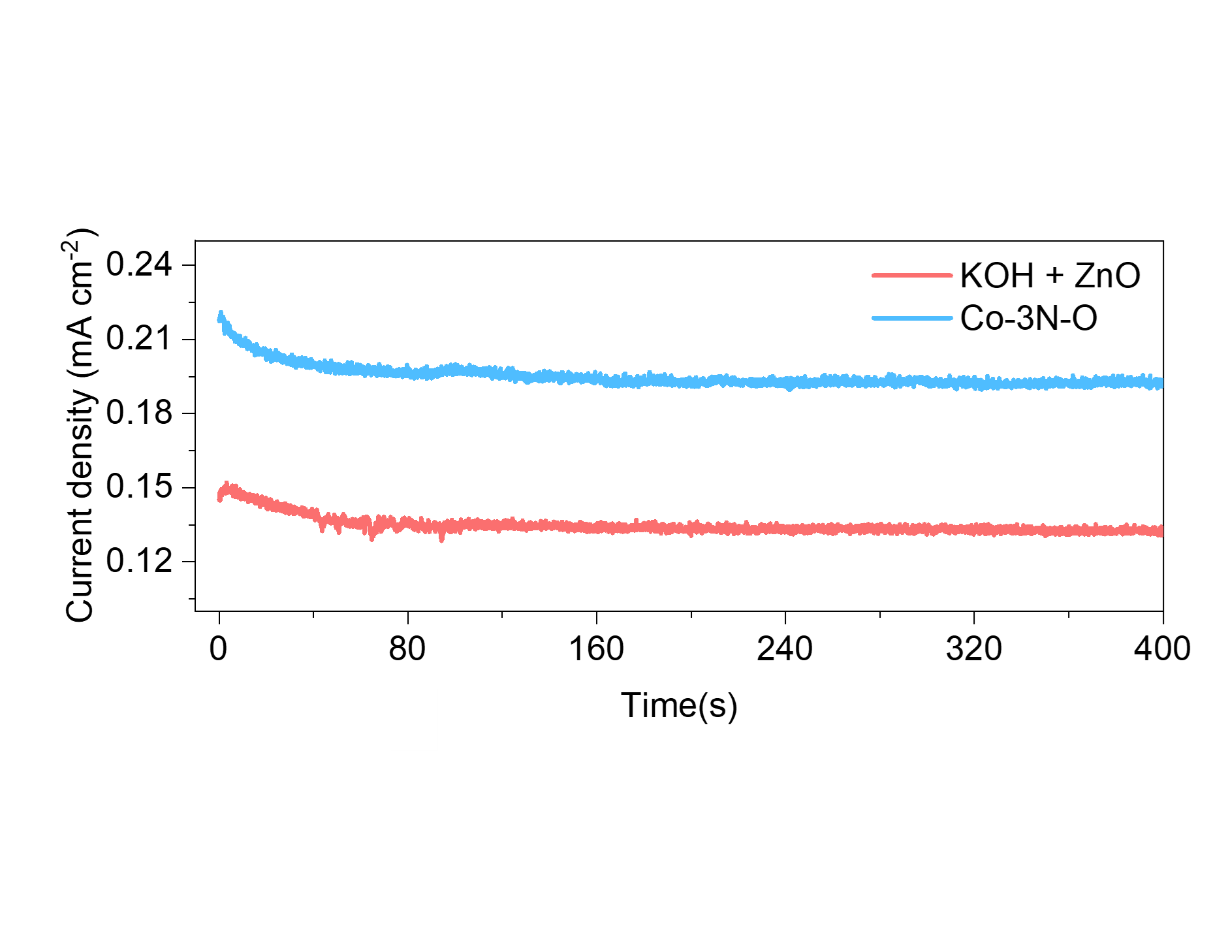


**Figure S12.** The current density of KOH + ZnO and Co-3N-O over 3000 s under constant potential.


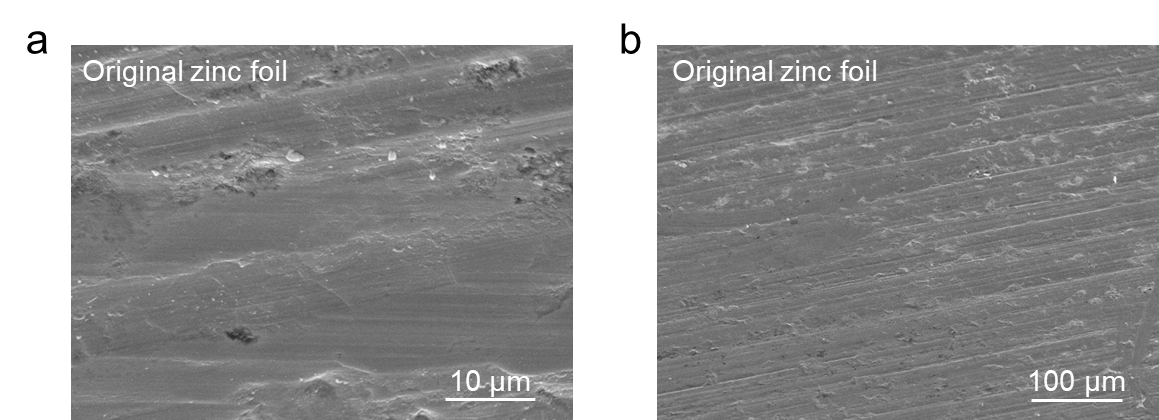


**Figure S13.** The original zinc foil surface at (a) 10 μm and (b) 100 μm scale.


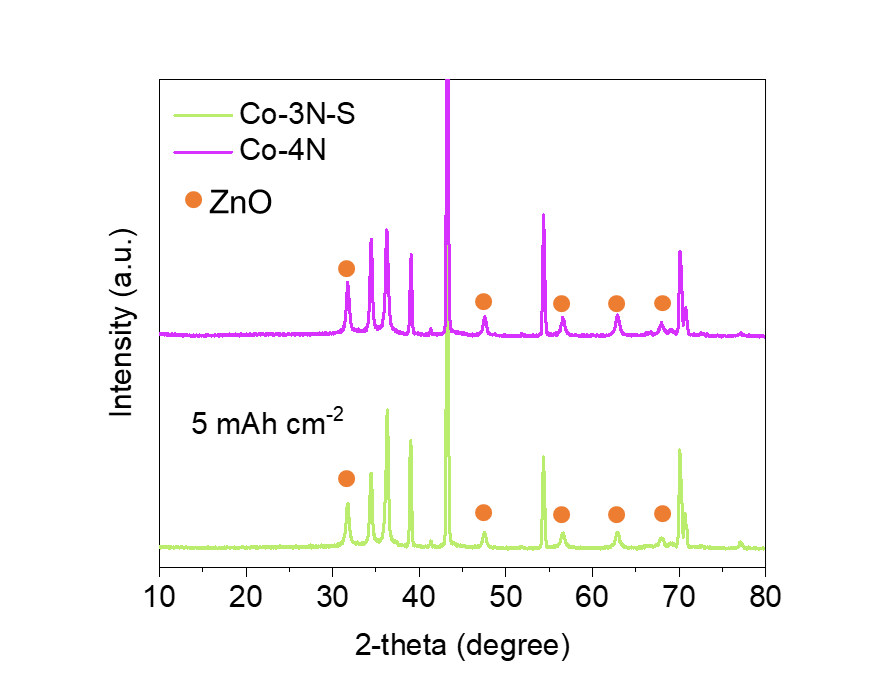


**Figure S14.** XRD spectra of the zinc anode of Co-3N-S and Co-4N electrolyte at 5 mAh cm^-2^.


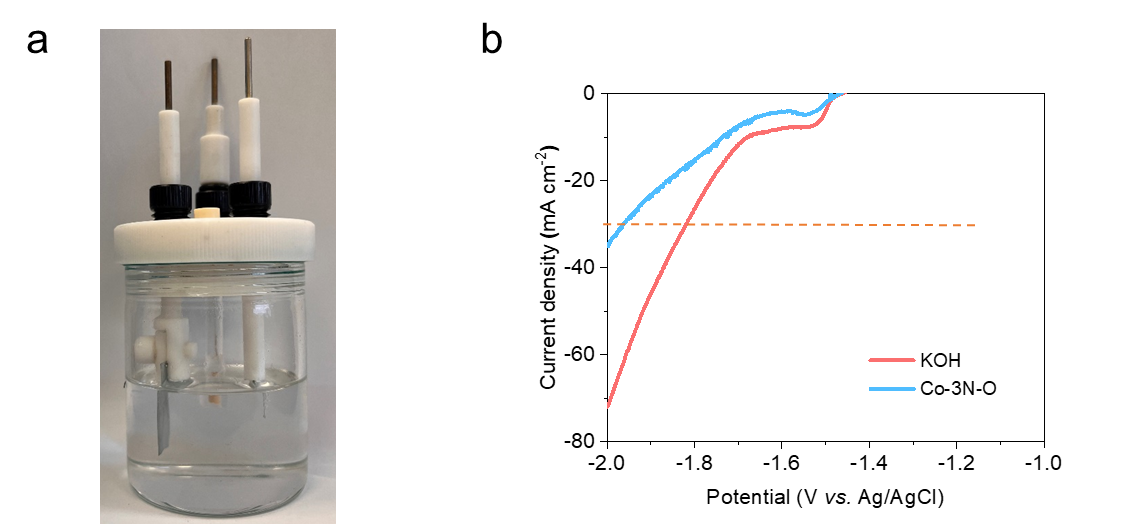


**Figure S15.** LSV curves of the KOH and Co-3N-O electrolyte systems.


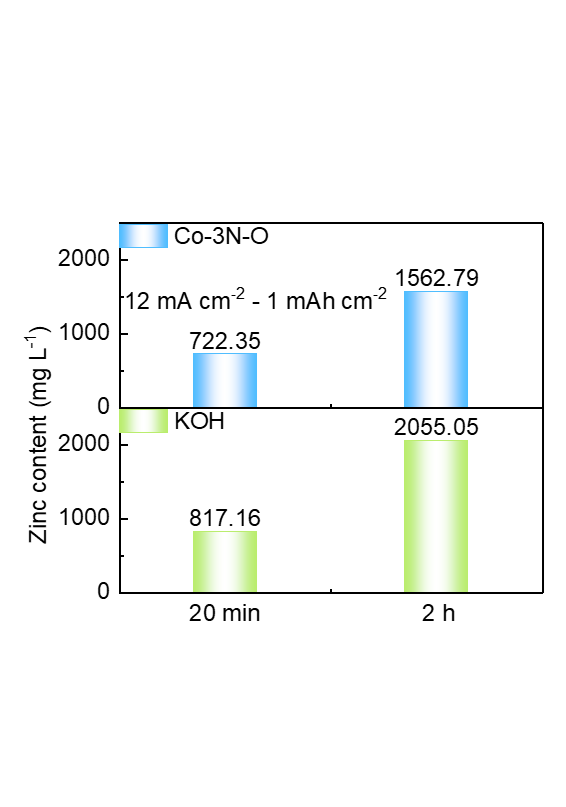


**Figure S16.** The zinc content of Co-3N-O and KOH electrolyte after cycling at 12 mA cm^-2^ - 1 mAh cm^-2^ at 20 min and 2 h (zinc electrode: 2 × 2 cm^2^).


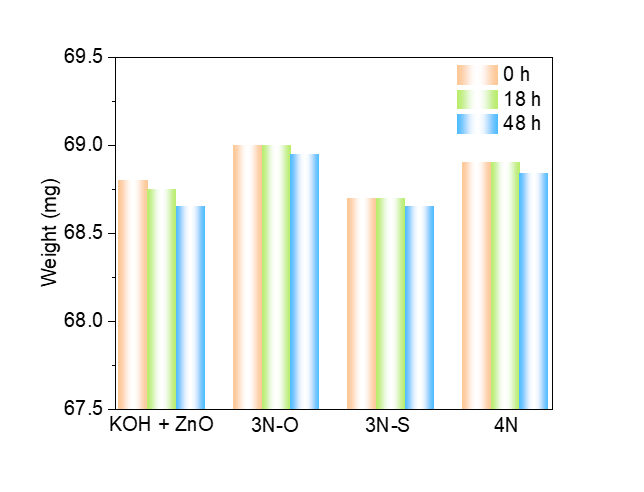


**Figure S17.** Anode weight measured before and after immersion in the electrolytes for 0, 18, and 48 h.


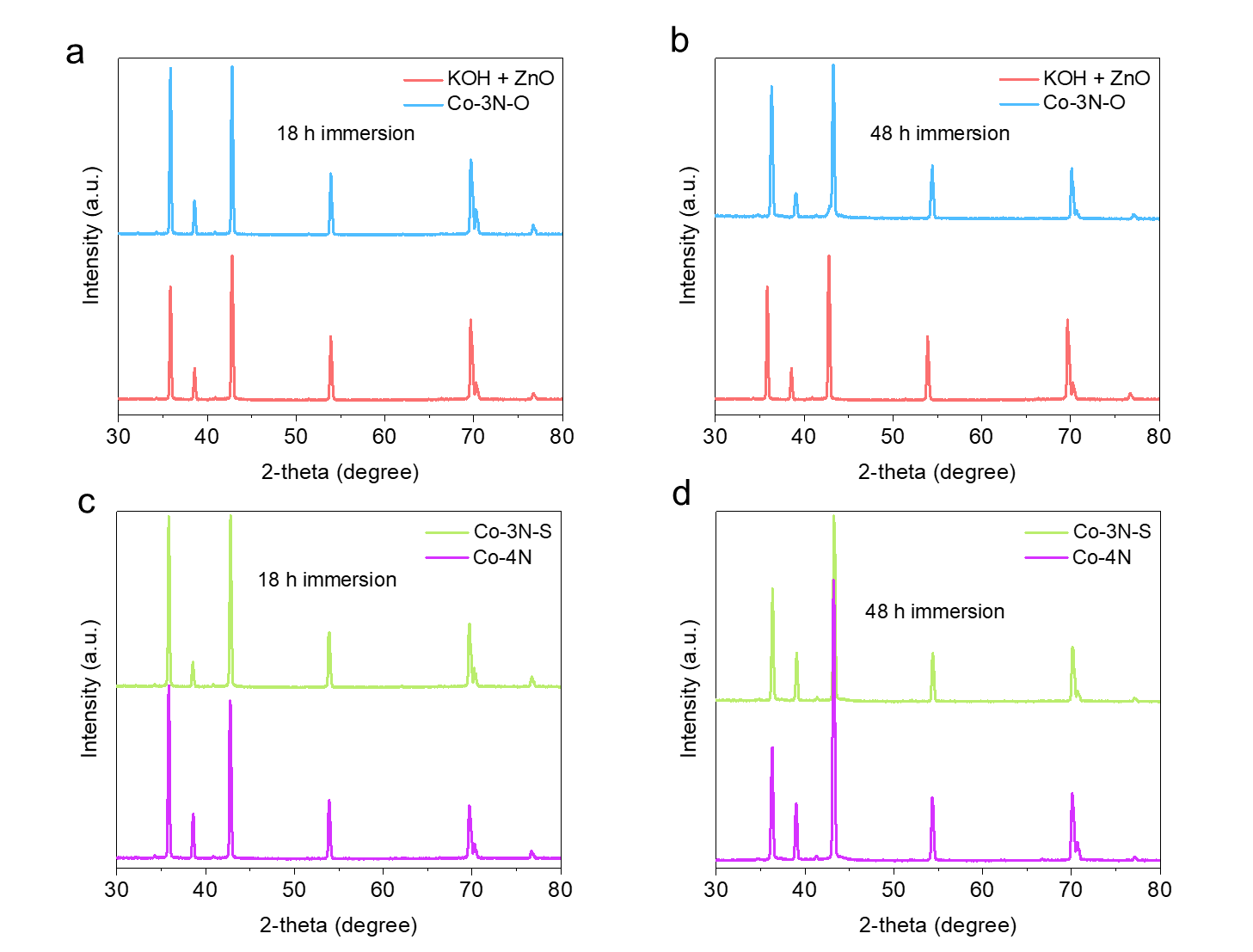


**Figure S18.** XRD spectra of zinc anode after immersing for (a) 18 h and (b) 48 h in KOH + ZnO and Co-3N-O electrolytes, and after immersing for (c) 18 h and (d) 48 h at Co-3N-S and Co-4N electrolytes.


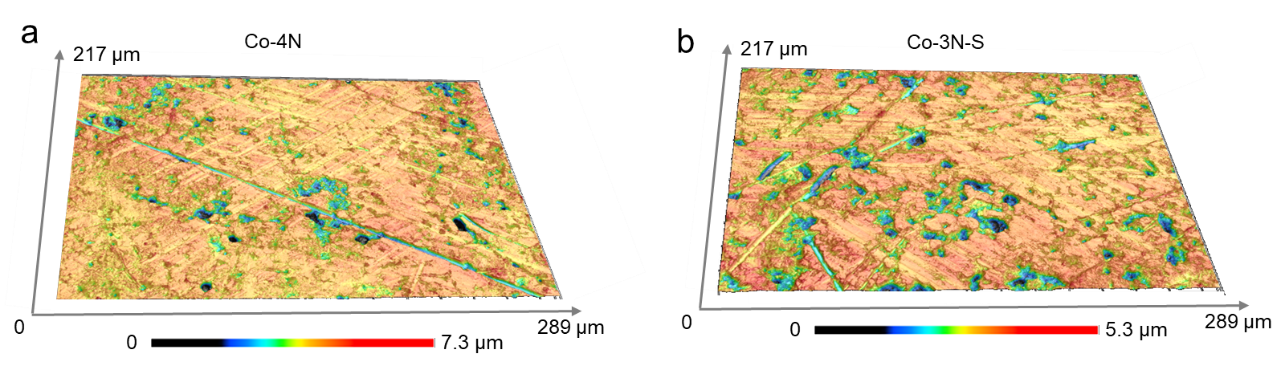


**Figure S19.** The CLSM 3D images of zinc anode after immersing for 48 h in (ab) Co-4N and (cd) Co-3N-S electrolytes.


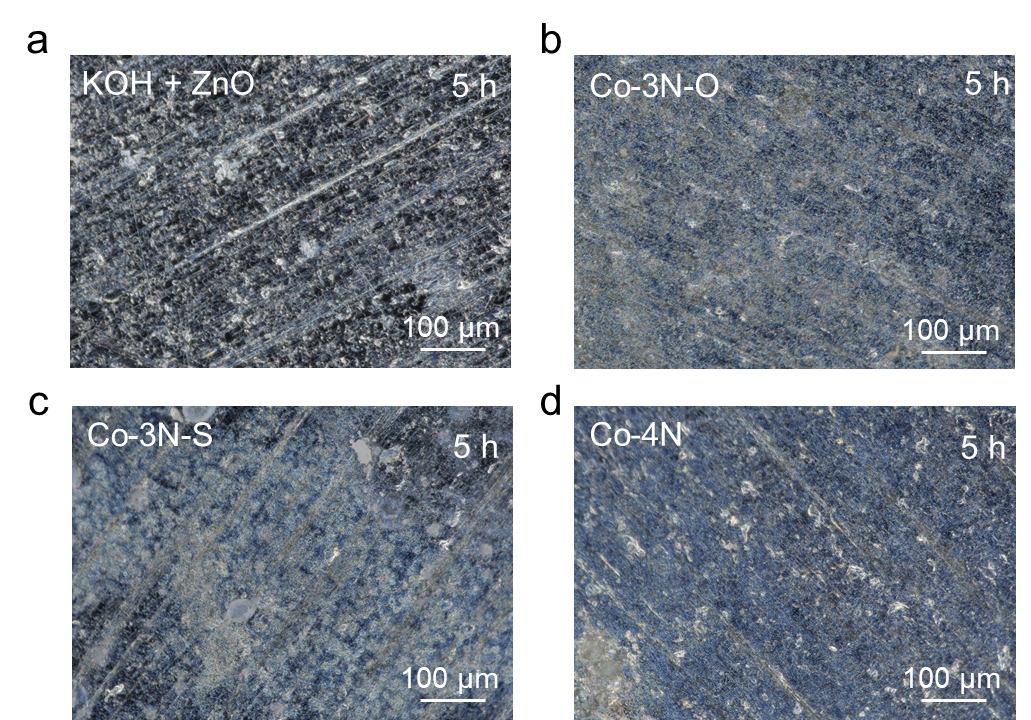


**Figure S20.** Zinc anode surface morphology after immersing for 5 h in (a) KOH + ZnO, (b) Co-3N-O, (c) Co-3N-S, and (d) Co-4N electrolyte.


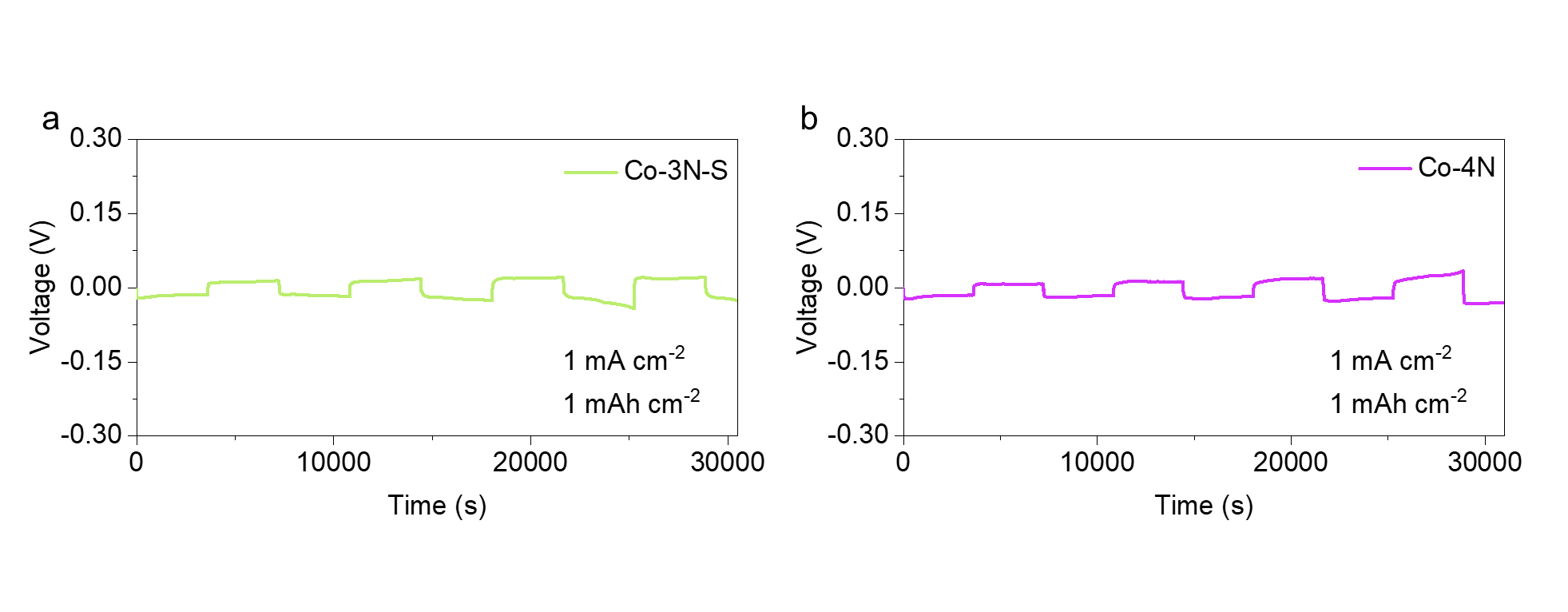


**Figure S21.** Cycling performance of (a) Co-3N-S and (b) Co-4N symmetric battery at 1 mA cm^-2^.


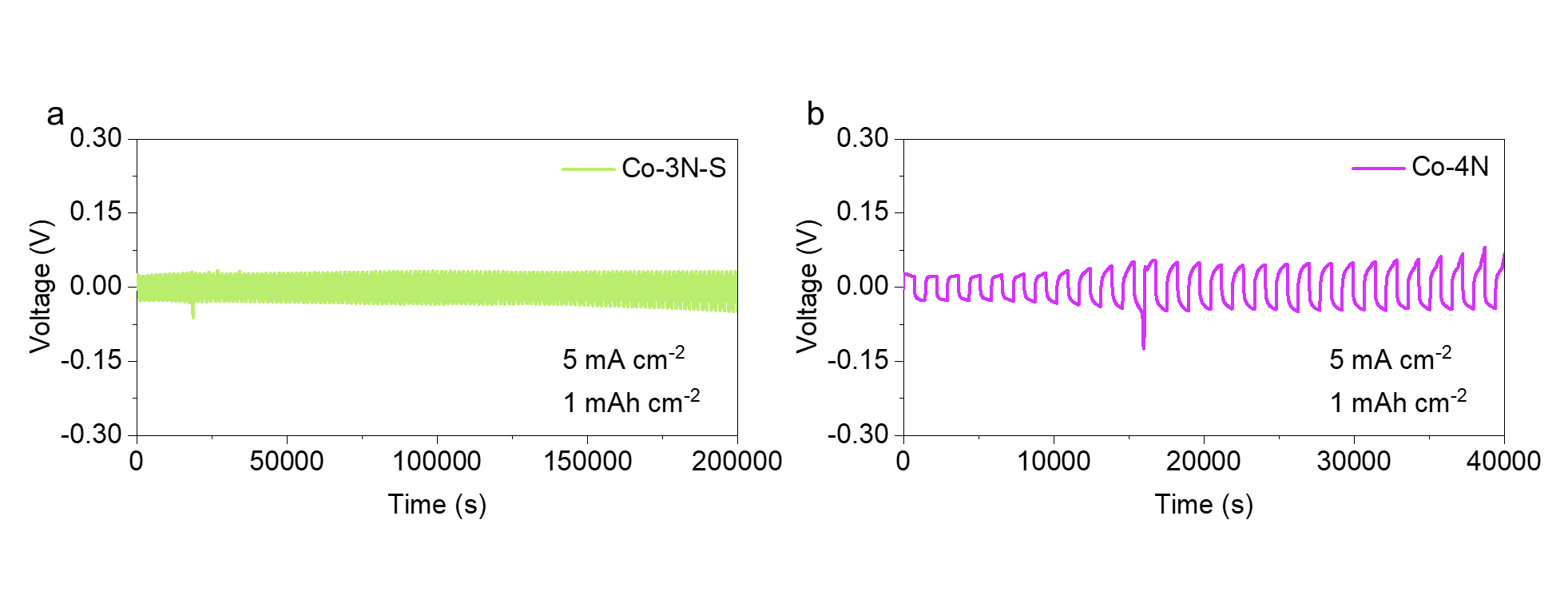


**Figure S22.** Cycling performance of (a) Co-3N-S and (b) Co-4N symmetric battery at 5 mA cm^-2^.


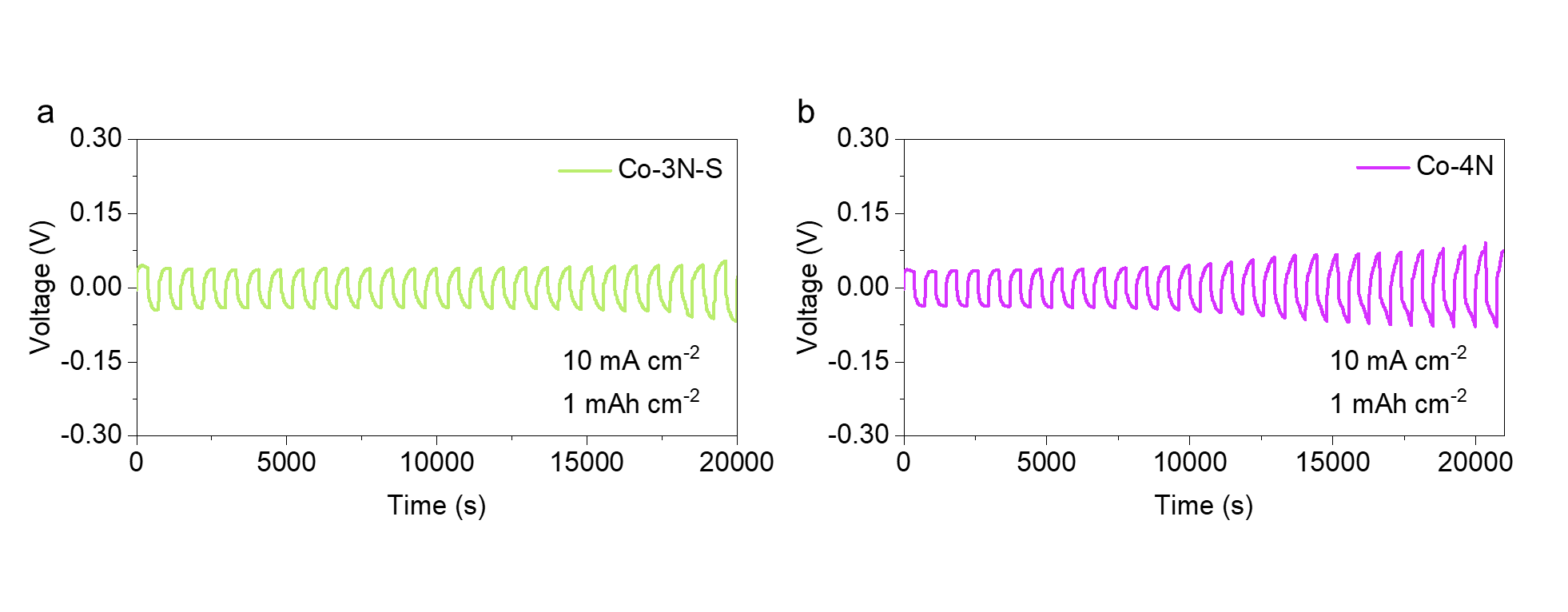


**Figure S23.** Cycling performance of (a) Co-3N-S and (b) Co-4N symmetric battery at 10 mA cm^-2^.


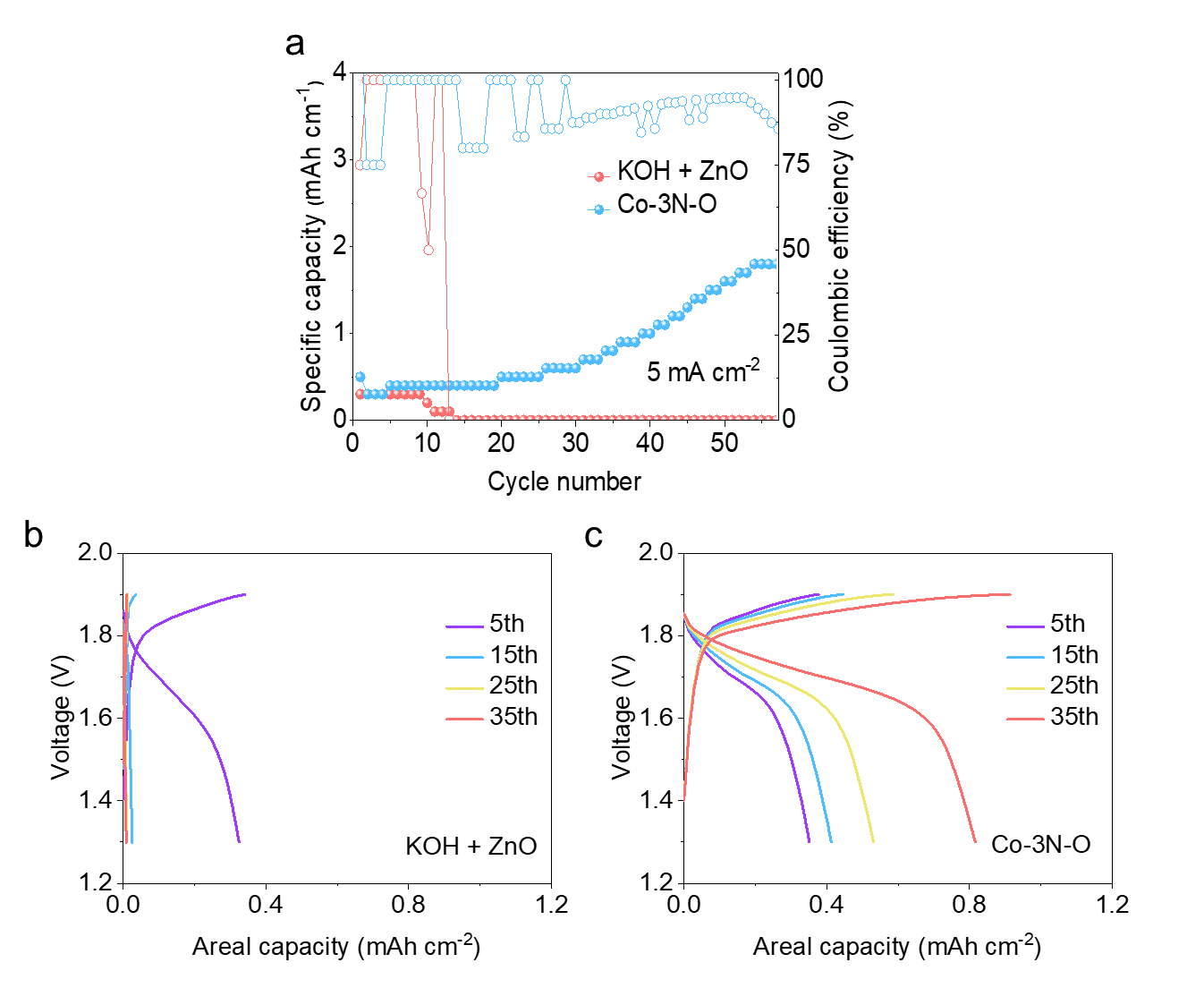


**Figure S24.** Cycling performance of full batteries assembled and the corresponding voltage-capacity profiles of (b) KOH + ZnO and (c) Co-3N-O at the current density of 5 mA cm^-1^.


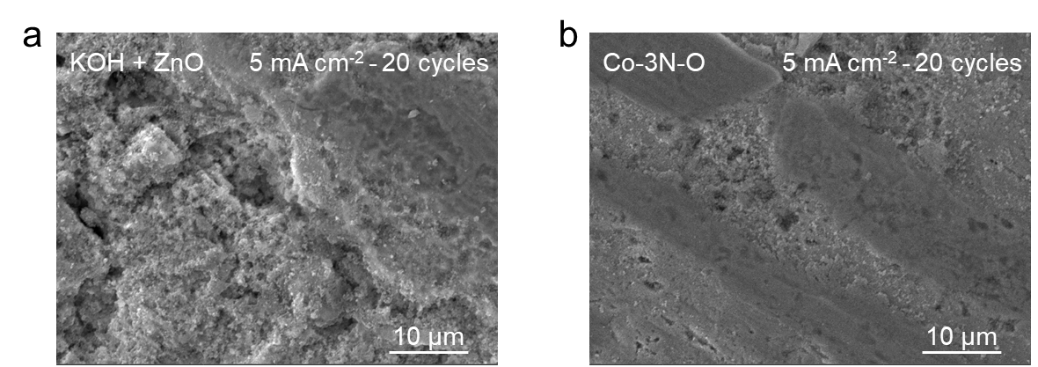


**Figure S25.** SEM images of the zinc anodes disassembled from 20-times-cycled zinc-nickel battery with (**e**) KOH + ZnO and (**f**) Co-3N-O electrolytes at 5 mA cm^-2^.


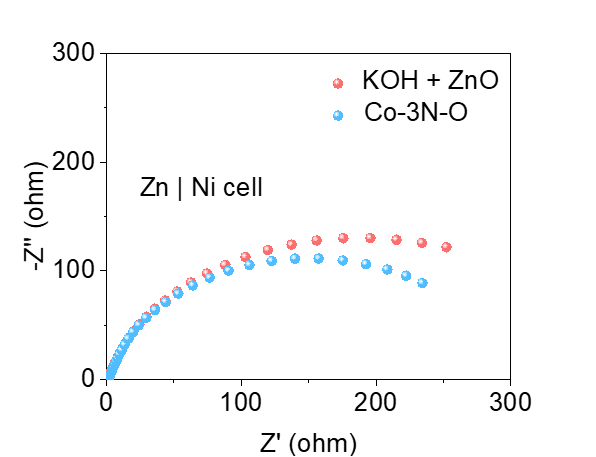


**Figure S26.** EIS curves of KOH + ZnO and Co-3N-O asymmetric battery.
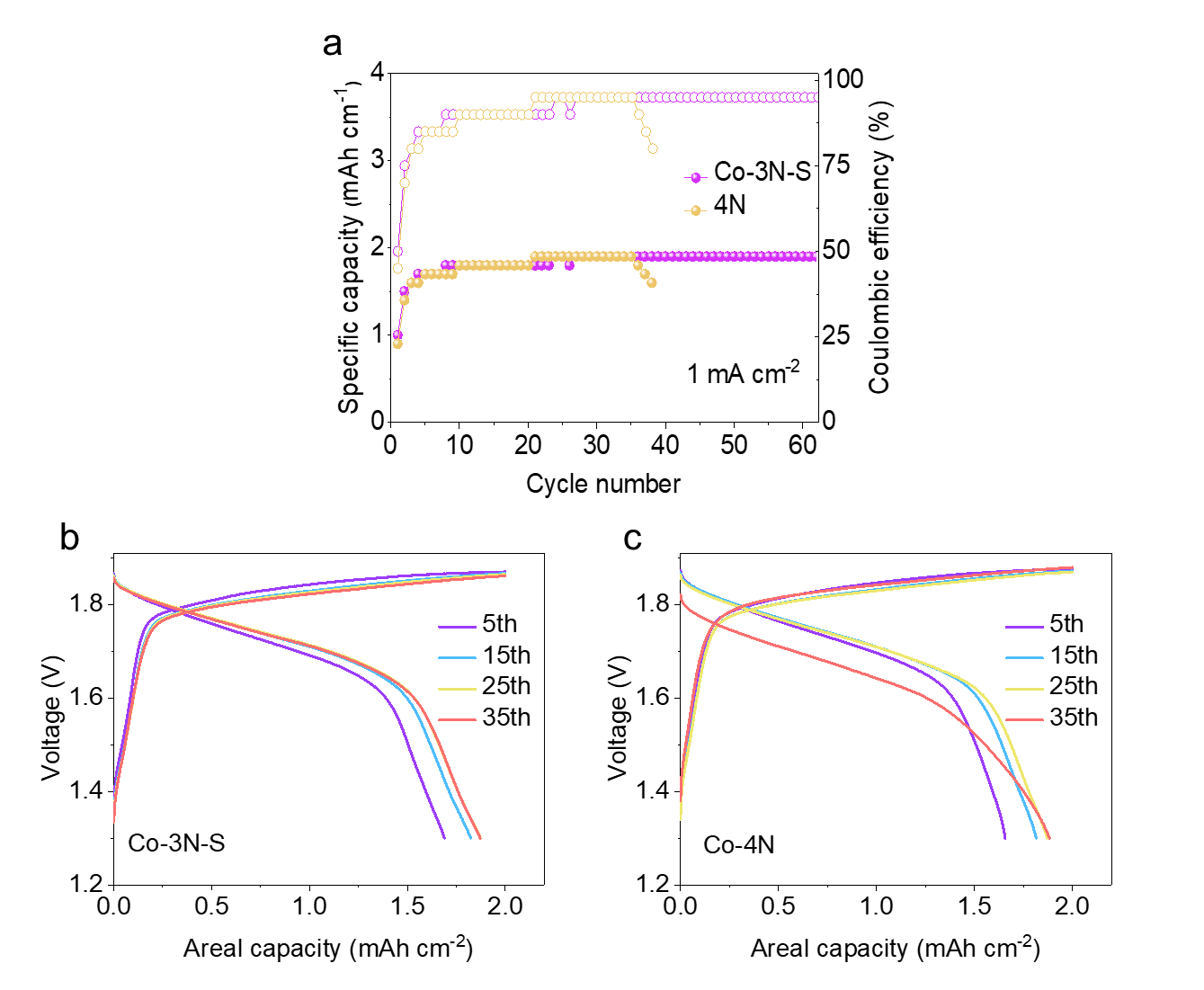


**Figure S27.** (a) Cycling performance of full batteries assembled and the corresponding voltage-capacity profiles of (b) Co-3N-S and (c) Co-4N at the current density of 1 mA cm^-1^.

**References**

[1] a) M. O. Cichocka, Z. Liang, D. Feng, S. Back, S. Siahrostami, X. Wang, L. Samperisi, Y. Sun, H. Xu, N. Hedin, H. Zheng, X. Zou, H. C. Zhou, Z. Huang, *J. Am. Chem. Soc.* **2020**, *142*, 15386-15395; b) Y. H. Zhong, Y. Wang, S. Y. Zhao, Z. X. Xie, L. H. Chung, W. M. Liao, L. Yu, W. Y. Wong, J. He, *Adv. Funct. Mater.* **2024**, *34*, 2316199.

[2] a) S. Stute, K. Gloe, K. Gloe, *Tetrahedron* **2005**, *61*, 2907-2912; b) S. B. Mane, C.-H. Hung, *New J. Chem.* **2014**, *38*, 3960-3972; c) R. Ambre, C.-Y. Yu, S. B. Mane, C.-F. Yao, C.-H. Hung, *Tetrahedron* **2011**, *67*, 4680-4688.

[3] Z. Zhao, R. Wang, C. Peng, W. Chen, T. Wu, B. Hu, W. Weng, Y. Yao, J. Zeng, Z. Chen, P. Liu, Y. Liu, G. Li, J. Guo, H. Lu, Z. Guo, *Nat. Commun.* **2021**, *12*, 6606.

[4] L. Hong, X. Wu, L. Y. Wang, M. Zhong, P. Zhang, L. Jiang, W. Huang, Y. Wang, K. X. Wang, J. S. Chen, *ACS Nano* **2022**, *16*, 6906-6915.

[5] D. M. R. de Rooij, *Anti-Corros. Methods Mater.* **2003**, *50*.
